# Catching the π-Stacks: Prediction of Aggregate Structures of Porphyrin

**DOI:** 10.1021/acs.jpca.4c05969

**Published:** 2024-11-09

**Authors:** Anna Elmanova, Burkhard O. Jahn, Martin Presselt

**Affiliations:** † Institute of Physical Chemistry, Friedrich Schiller University Jena, Helmholtzweg 4, 07743 Jena, Germany; ‡ Leibniz Institute of Photonic Technology (IPHT), Albert-Einstein-Str. 9, 07745 Jena, Germany; § SciClus GmbH&Co. KG, Moritz-von-Rohr-Str. 1a, 07745 Jena, Germany; ∥ Center for Energy and Environmental Chemistry Jena (CEEC Jena) Friedrich Schiller University Jena, Philosophenweg 7a, 07743 Jena, Germany

## Abstract

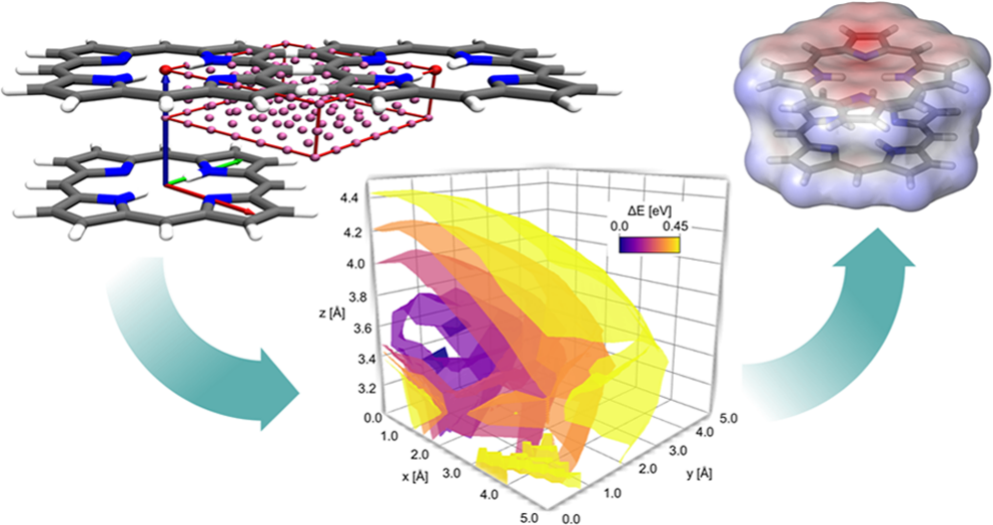

π–π interactions decisively shape the supramolecular structure and functionality of π-conjugated molecular semiconductor materials. Despite the customizable molecular building blocks, predicting their supramolecular structure remains a challenge. Traditionally, force field methods have been used due to the complexity of these structures, but advances in computational power have enabled ab initio approaches such as density functional theory (DFT). DFT is particularly suitable for finding energetically favorable structures of dye aggregates, which are determined by a large number of different interactions, but a systematic aggregate search can still be very challenging due to the large number of possible geometries. In this work, we show ways to overcome this challenge. We investigate how finely translational and rotational lattices must be structured to identify all energetic minima of π-stack structures, focusing on porphyrins as a prototype challenge. Our approach involves single-point DFT calculations of systematically varied dimer geometries, identification of local energy minima, hierarchical grouping of geometrically similar structures, and optimization of the energetically favorable representatives of each geometric family. This ab initio method provides a general framework for the systematic prediction of aggregate structures and reveals geometrically diverse and energetically favorable dimers.

## Introduction

Organic materials are being intensively researched and developed and are already being used in a variety of devices ranging from solar cells to light-emitting diodes.^1^ In addition to the properties of the individual molecules, the intermolecular interactions and thus the supramolecular or aggregate geometry have a particularly strong influence on the optical and electronic properties, see H- and J-aggregates in liquid crystals or supramolecular variations of energy layers and open circuit voltages in solar cells.^2^ The geometries of dye aggregates can be influenced by varying the molecular structure (e.g., substitution of side groups of the dyes^3,4^) as well as the parameters of material formation.^5^ Especially for synthetic chemistry, it would be invaluable if aggregate geometries could be predicted routinely and quickly for arbitrary molecular structures to be synthesized. Since the predominance of different aggregate geometries also depends on the processing parameters, an overview of the energetically favored aggregates is particularly important, while their exact energetic sequence is of secondary importance.

Hence, fast ab initio predictions of supramolecular structures, such as those found in aggregates,^6,7^ unit cells of crystalline,^8−10^ or even polymorphic materials,^11−13^ would significantly advance the targeted development of functional materials.^14,15^ Various computational approaches, including the hybrid density functional theory (DFT)/molecular mechanics method,^10^ periodic second-order Møller–Plesset perturbation theory,^12^ and traditional DFT calculations,^10,16^ have been validated for their efficacy in determining the properties of given complex molecular systems.

However, the structures provided by the established optimization algorithms, combined with all of the aforementioned highly accurate ab initio methods, are strongly dependent on the starting geometries. Ideally, starting geometries are chosen that are close to all energetic minima of the energy hypersurface of aggregate structures. Several approaches have been developed to identify them:

- evolutionary algorithm,^17^
- global swarm optimization,^8,18^
- random search,^19^
- simulated annealing,^20^
- meta-dynamics,^21^ and
- systematic searches^14^

**Figure 1:**
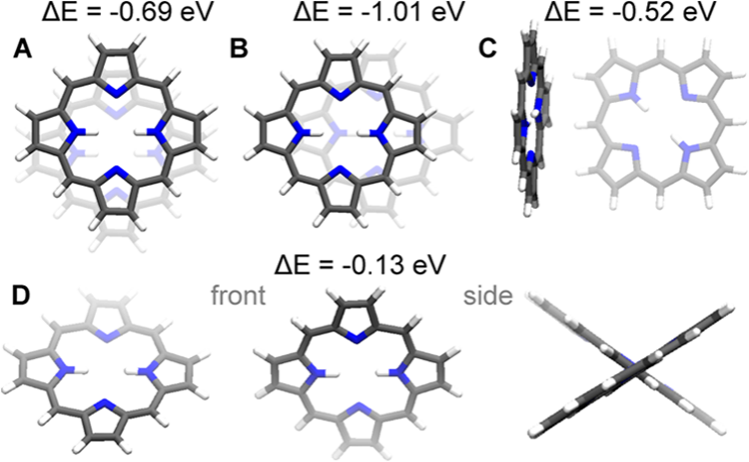
Porphyrin dimer structures: Sandwich (A), parallel-displaced (B), T-shape (C), and displaced X-shape (D). Reproduced from ref (14). Copyright 2024 John Wiley and Sons. Reproduced from ref (14). Copyright 2024 John Wiley and Sons.

The systematic approach, by definition, treats all dimers within the given parameter space, and is thus not prone to wasting computational time on single potential energy minima, as might happen in meta-dynamics or simulated annealing, or missing structures due to limitations in chemical intuition. However, it is often necessary to explore numerous aggregate geometries,^13,22^ which is typically done by force field methods. Therefore, even the systematic approach can miss significant local minima, as exemplified by the porphyrin sandwich dimer (see structure A in Figure 1), where crucial London dispersion interactions were not adequately captured by the force fields employed, leading to an underestimation of their binding energy.^14^ However, other porphyrin dimers reported in the literature, where different interactions play an important role, were correctly identified.^14^ Given the diverse interactions among porphyrins, predicting their dimer structures is an ideal challenge for quantum chemistry.

With ever-increasing computational power, the question arises as to what resources and strategies the systematic search for dimer structures using ab initio methods is possible. Previous work has shown that the systematic variation of the position of a second porphyrin, together with the consideration of different rotations of the molecules in a porphyrin dimer relative to each other, quickly requires the calculation of several billion structures.^14^ In this work, we focus on the dimers that have proven most difficult to predict by force field methods in the literature - the π-stacks (Figure 1A,B). We focus on coplanar initial structures and investigate how fine the spatial positional variations of the relative center of mass coordinates of two porphyrins must be in order for DFT-based geometry optimizations to provide all local minima structures based on the automatically and systematically generated initial structures.

## Method Details

Quantum chemical structure optimizations along with the calculations of electrostatic potentials were carried out using density functional theory (DFT) as implemented in the GPU-accelerated program TeraChem^23,24^ with the lanl2dz, ahlrichs_pvdz, and 6-311+g basis sets^25^ and functionals B3LYP^26^ and CAM-B3LYP,^27^ which have shown to yield reasonable geometries, energies, electron density and electrostatic potential distributions and spectra.^4,28^ These density functionals are also known for their precision in calculation of molecular energies.^29^ Electrostatic potentials were plotted on the solvent-accessible surface^30,31^ for solvent probe of radius 1.4 Å.^31−33^

## Results and Discussion

**Figure 2:**
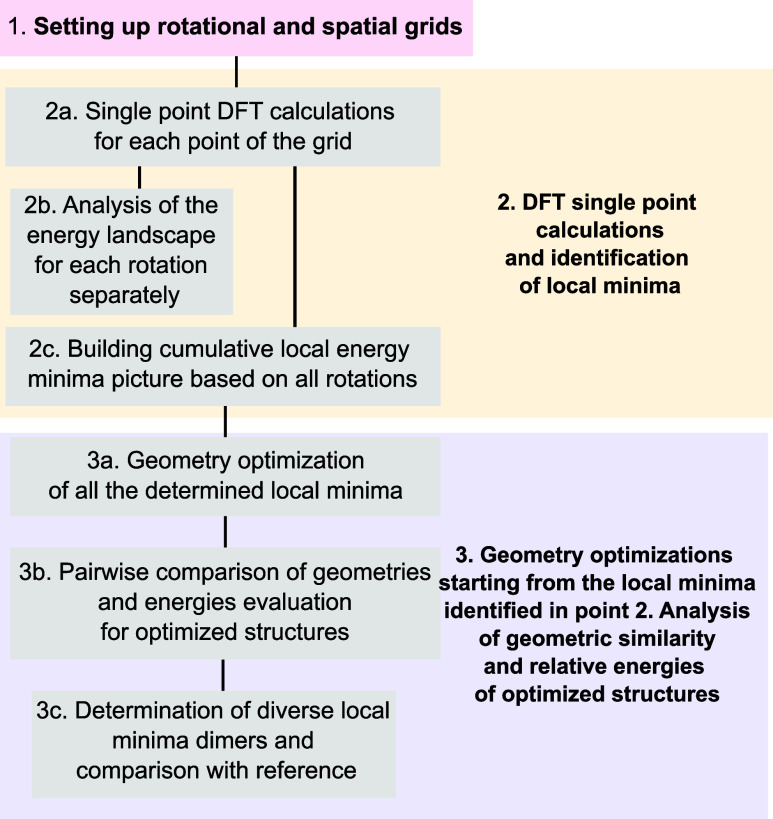
Colored blocks represent the main structural units of the aggregate search, while the subprocesses delineate the algorithmic steps or computational procedures.

**Figure 3:**
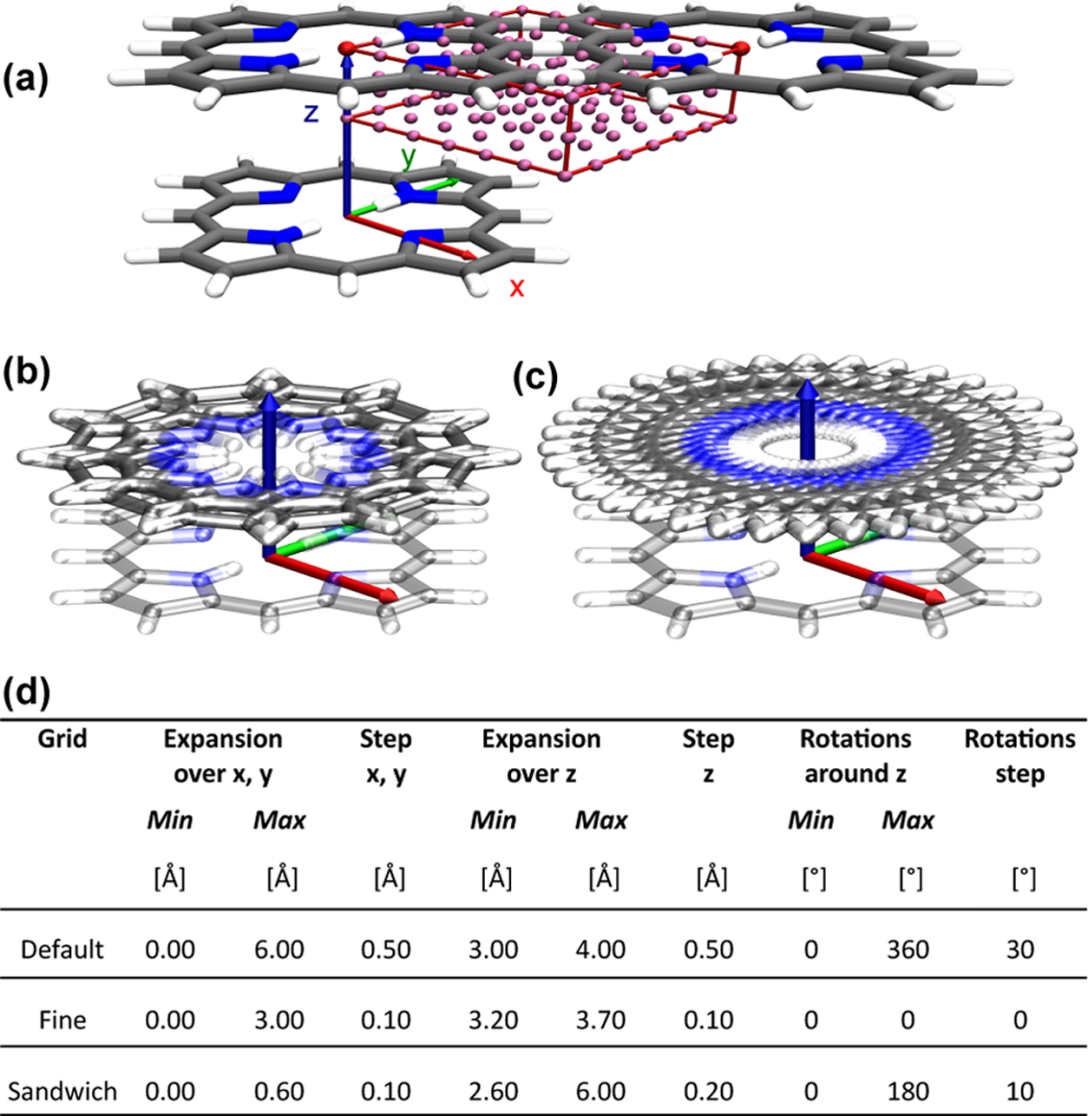
Spatial and rotational grids used in the present work to determine aggregates. Panel (a): Spatial grid with expansion to 6 Å over x and y axes and steps of 0.5 Å. Panel (b): Coarse rotation grid with rotation step around *z* axis of 30°. Panel (c): Fine rotation grid with steps of 10°. Panel (d): Details of the grids used in this work.

The generally applicable methodology employed in this study, illustrated in Figure 2, involves setting up translational and rotational grids, DFT single-point energy calculations for each systematically varied position and rotation of the second porphyrin. Figure 3 shows one spatial grid and two differently coarse rotational grids. In Figure 3d all the grids employed in the study are listed. Following the identification of local minima within these grids, corresponding geometries undergo geometry optimization until reaching energetically favorable structures. To assess if the optimization algorithms lead to convergence of different dimer starting configurations into identical ones, we conduct similarity searches. These searches utilize the root-mean-square deviation (RMSD) metric^34^ together with hierarchical clustering, an unsupervised machine learning algorithm.^35^ This approach facilitates the identification of geometrically distinct and energetically favorable dimer configurations.

### Generation of Initial Aggregate Structures

The algorithm used in this study is systematic, starting with the placement of the molecule in the center of the coordinate system (Figure 3). Subsequently, the initial molecule is duplicated, with rotations and translations applied. Rotations are constrained to the *z*-axis, taking advantage of the symmetry of the porphyrin molecule.

The translations considered in the *z*-direction are confined to a range starting at twice the van der Waals distance as the minimum, and extending no further than this minimum distance plus 2.0 Å,^14^ encompassing the typical range observed in molecular π-stacks (Figure 3).^36^ The variations in *x*- and *y* directions are restricted from 0 (no lateral shift) to two-thirds the molecular length in the corresponding direction (Figure 3). This general approach enables a systematic exploration of the potential energy hypersurface. The details about the extensions and steps of the “default”, “fine”, and “sandwich” grids employed within this work are shown in Figure 3. The “default” grid has in total 6084 points, the “fine” 174, and “sandwich” grid has 1944 points.

### Local Minima from Single-Point Calculations

The energy of all created dimers is determined via DFT single-point calculations as implemented in the program TeraChem.^23,37^ To study the dependence of local minima position on the basis set, we used lanl2dz_ecp^38^ and the valence double-ζ basis set ahlrichs_pvdz with polarization functions for all atoms as introduced by Ahlrichs and co-workers.^39^ Further, we used the hybrid density functional B3LYP and its range-corrected version CAM-B3LYP.^40^ All calculations were performed in vacuum and using the conductor-like screening model COSMO for perfect screening of surface charges.^27^ For reasonable description of dispersion interactions, we used Grimme’s D3-dispersion correction throughout our work. Unless otherwise stated, the following discussions refer to the combination B3LYP/ahlrichs_pvdz/COSMO/D3, where the COSMO model is used in the conductor limit to mimic favorable interactions with the environment, which are expected if the dimers would be part of thermodynamically stable solids.

**Figure 4:**
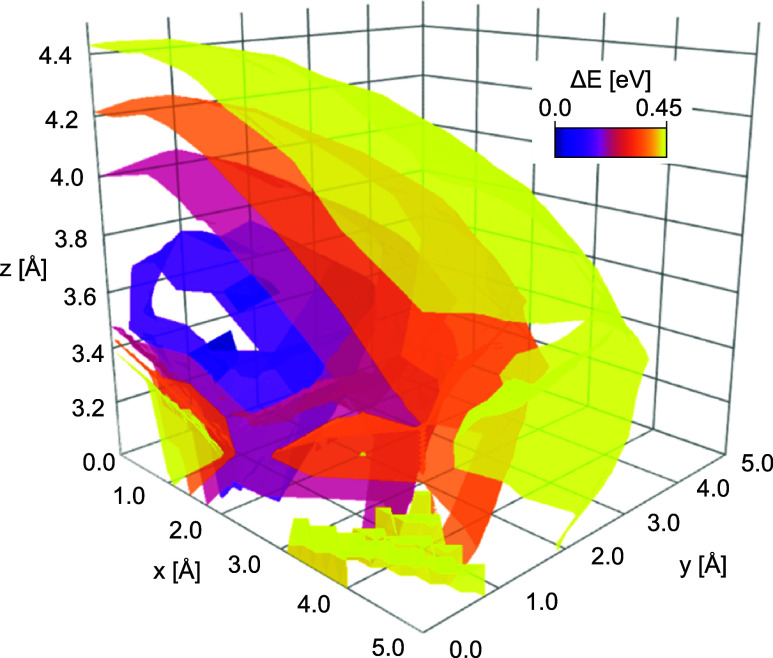
Energy isosurfaces (smoothened via cubic function) for the dimer search without consideration of rotations. The lowest minima in energy are enveloped separately by the inner, dark-blue surface.

**Figure 5:**
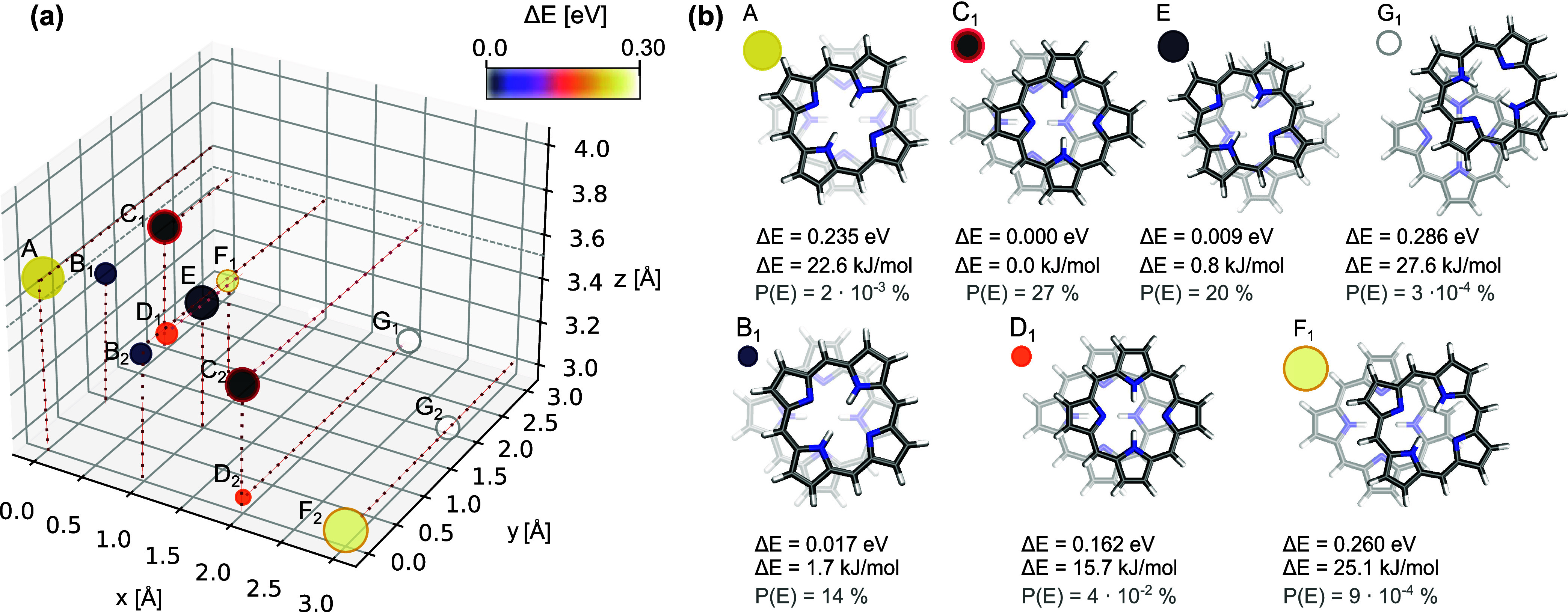
Panel (a): Distribution of the local energy minima considering all rotations; The size of each scatter shows how often local minima were found at the considered point in space for all rotation angles. Structures with global minima in the energies are denoted with red contours. Panel (b): Determined dimer structures in their local minima rotations with corresponding relative energies with respect to the global minima over all rotations and translations (C), as well as the probability of their occurrence according to Boltzmann distribution.

Figure 4 shows the energy isosurfaces for dimers with no rotations applied to the second porphyrin. For these nonrotated dimers, two distinct local energy minima positions are revealed through the regions enveloped by the dark-blue isosurfaces in Figure 4. The centers of these regions are the points (2.0, 0.0, 3.5) and (0.0, 2.0, 3.5). Figure 5a depicts local energy minima positions for all rotation angles. The sizes of the circles in Figure 5a scale with the number of local minimum positions found at the corresponding point for all considered rotations. The color of the circles indicates the energy of the energetically most favorable dimer in each group (labeled A-G). Structures that possessed symmetric counterparts were designated with numerical identifiers (e.g., C_1_ and C_2_). The local favorite structures are shown in Figure 5b.

To determine the probability *P*_*i*_(*E*) of each conformer to be observed at room temperature, the Boltzmann distribution was used:
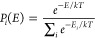
with *T* = 293 K, *k* being the Boltzmann constant and summation is done over all dimers identified as local minima (indexed *i*; the dimers considered are rotated around the *z* axis by 0, 30, 60, 90° and their translations are bound to a maximum of 3 Å along *x* and *y* axes). These probabilities are shown in Figure 5b.

In the first coarse search using the default grid, energetically favorable dimers were found in the range of 0 Å–3 Å along the *x* and *y* axes. The most favorable dimers are those that are slightly slip-stacked (Figure 5B,C,E), while those that are not laterally shifted (sandwich structure) or are shifted by more than 2.5 Å in *x*, *y*, or *x*, *y* directions (F, G) are energetically less favorable. Full information on the geometric and energetic properties of the dimers is presented in the SI. The local minima identified upon rotation indicate that the rotated structures are generally more favorable than their nonrotated counterparts. Furthermore, the extensive set of single-point calculations discovers the “sandwich” structure as a local minimum, which was not obtained by the previous force field based calculations.^14^ To refine the identified local minima structures, geometry optimization was performed for all applied rotations (0, 30, 60, 90°), as discussed below.

### Convergence to Local Minima via Geometry Optimization

**Figure 6:**
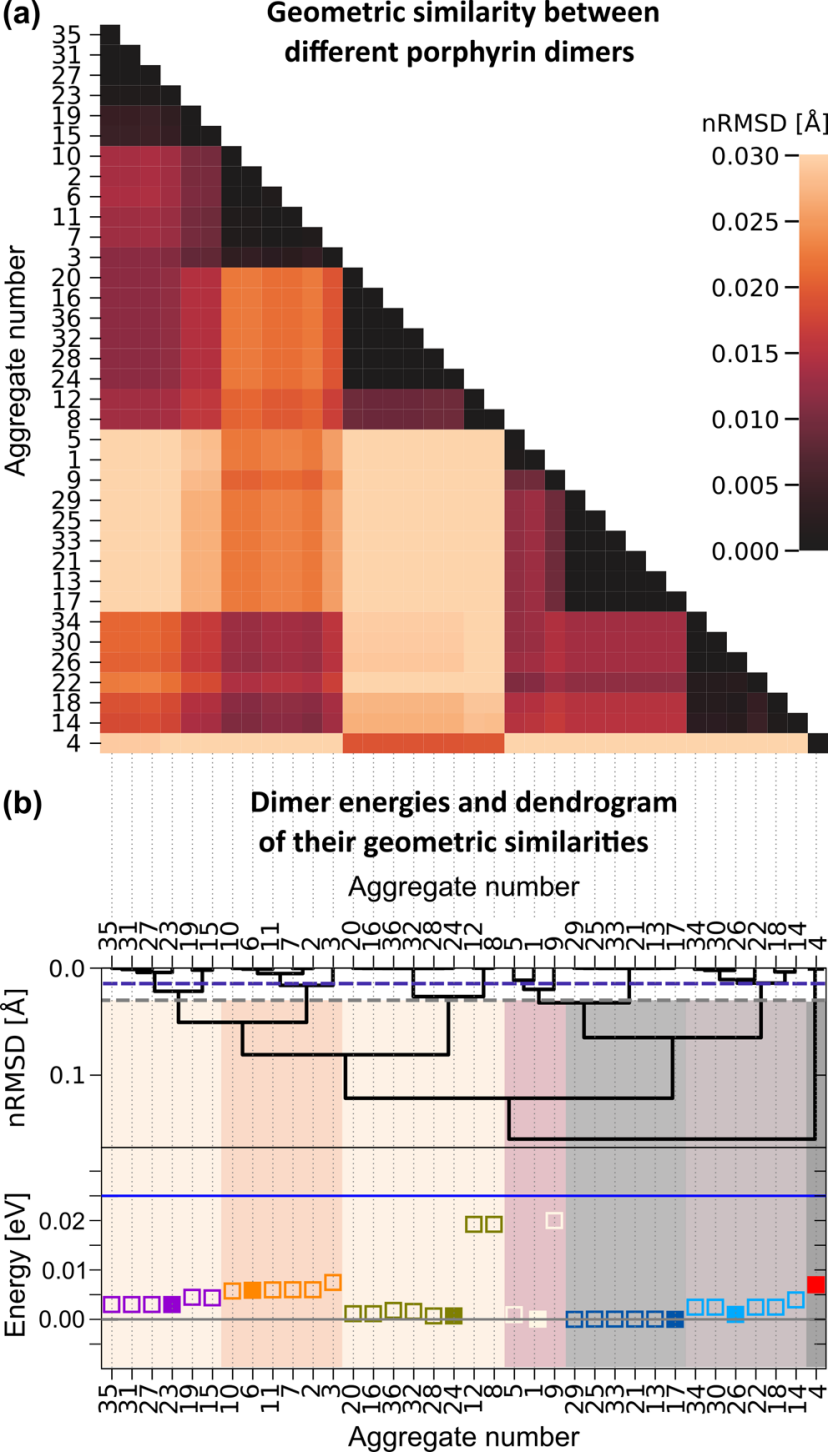
Energetic and geometrical comparison of structurally optimized dimers (B3LYP/ahlrichs_pvdz/COSMO). Panel (a): Heatmap of pairwise *n*RMSD values for all dimer structures. Panel (b): Dendrogram of the hierarchical clustering of the optimized dimers using pairwise *n*RMSD values shown in the heatmap above. *n*RMSD thresholds used to discuss geometric families are marked by dashed horizontal lines (0.03 and 0.015 Å; gray and blue dashed lines, respectively). The lower panel shows the corresponding dimer energies (geometric families for a *n*RMSD threshold of 0.03 Å have same colors; The minimum in each family is marked with a filled symbol). The blue horizontal line indicates an energy of 25 meV, which represents the thermal energy available at room temperature.

Figure 6 shows energies and geometric similarity data obtained after relaxing the geometries of the dimers, which were previously identified as local minima based on single-point calculations. The similarity is analyzed by calculating normalized pairwise root-mean-square deviations (*n*RMSDs) between the atomic positions of two different dimers (Figure 6a). In this normalization process, the RMSD values are divided by the number of atoms, which is supposed to facilitate the comparison of RMSD values of similarity analyses of differently sized systems. In the heatmap of the RMSDs in Figure 6c, dimers are ordered by their *n*RMSD values using hierarchical clustering, which is particularly effective for analyzing molecular structural similarities.^41^ In this study, we use agglomerative clustering, a bottom-up approach in which each data point is first treated as a single cluster. Subsequently, the nearest clusters are progressively merged into larger clusters until a single all-encompassing cluster remains. The details of the selection of the clustering method and the comparison with the results of k-means clustering can be found in SI, Section 3.

The dendrogram in Figure 6b illustrates the similarity relationships between the different dimers and two *n*RMSD threshold values used to discriminate similarity and dissimilarity are shown (dashed lines). For a *n*RMSD threshold of 0.03 Å (geometrically similar dimers: *n*RMSD < 0.03 Å; distinct dimers: *n*RMSD > 0.03 Å) we obtain seven unique families, which are color-coded in energy comparison figure in Figure 6b showing the energies of each geometrically optimized dimer.

**Figure 7:**
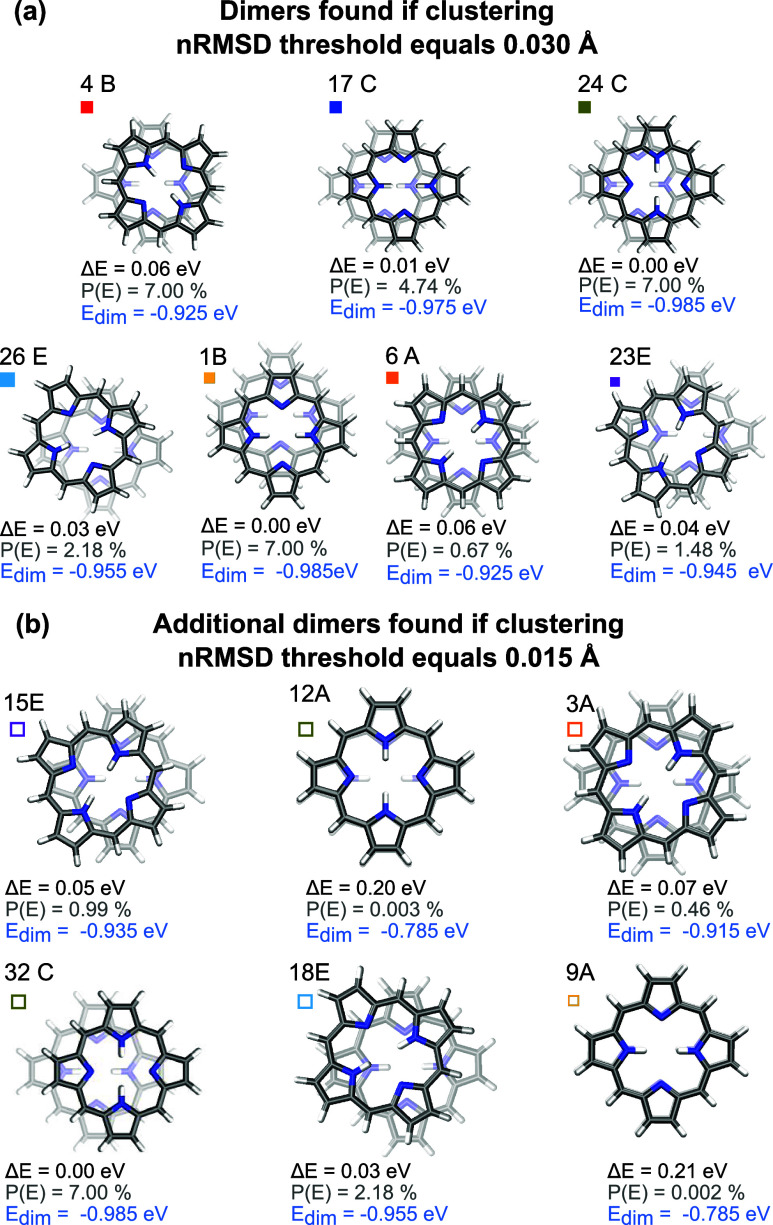
Energetically most favorable representatives of each geometric family. Panel (a): Dimers found with *n*RMSD threshold of 0.03 Å according to dendrogram shown above. Panel (b): Additional dimers found with a *n*RMSD threshold of 0.015 Å.

Representative dimer structures of these families are shown in Figure 7. Dimer 17 represents the slip-stacked dimer reported in literature (see dimer B in Figure 1). However, the analog dimers with the second porphyrin displaced along the direction defined by the two nonprotonated nitrogen (dimer 1) or rotated by 90° (dimer 24; global minimum), are energetically more favorable. We assume that the accuracy of these energy differences is better than 0.015 eV, what is found for the root-mean-square-deviation of noncovalent dimerization energies in benchmark sets containing systems that are sensitive to dispersion interactions.^29^ Structures 4, 14, 6, 19 represent dimers with initial 30 and 60° rotation, which rotate until 45° upon structure optimization.

The dimerization energy is calculated as


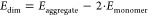
where the energy of the monomer (*E*_monomer_) is determined for the optimized porphyrin. Dimerization energies are denoted in blue in Figure 6c. In comparison with the nonrefined dimers (Figure 5), the optimized structured showed much less divergence in energy values and revealed that not 30 or 60° structures are energetically favorable, but 45° rotated ones. The structures initially rotated on these angles converged to 45°, while the 0 and 90° rotated structures only translated upon refinement.

A second *n*RMSD threshold of 0.015 Å was used, leading to the emergence of five new families, as indicated by the dendrogram in Figure 6b (the blue dashed line represents the new threshold). The energetic properties of the favorable instances within each of these new families are illustrated in Figure 7b. Notably, the nonrotated “sandwich” dimer was identified as distinct and the most favorable within a new family. This result highlights that the algorithm presented in this paper enables the identification of crucial aggregates not discovered through force field searches before.

**Figure 8:**
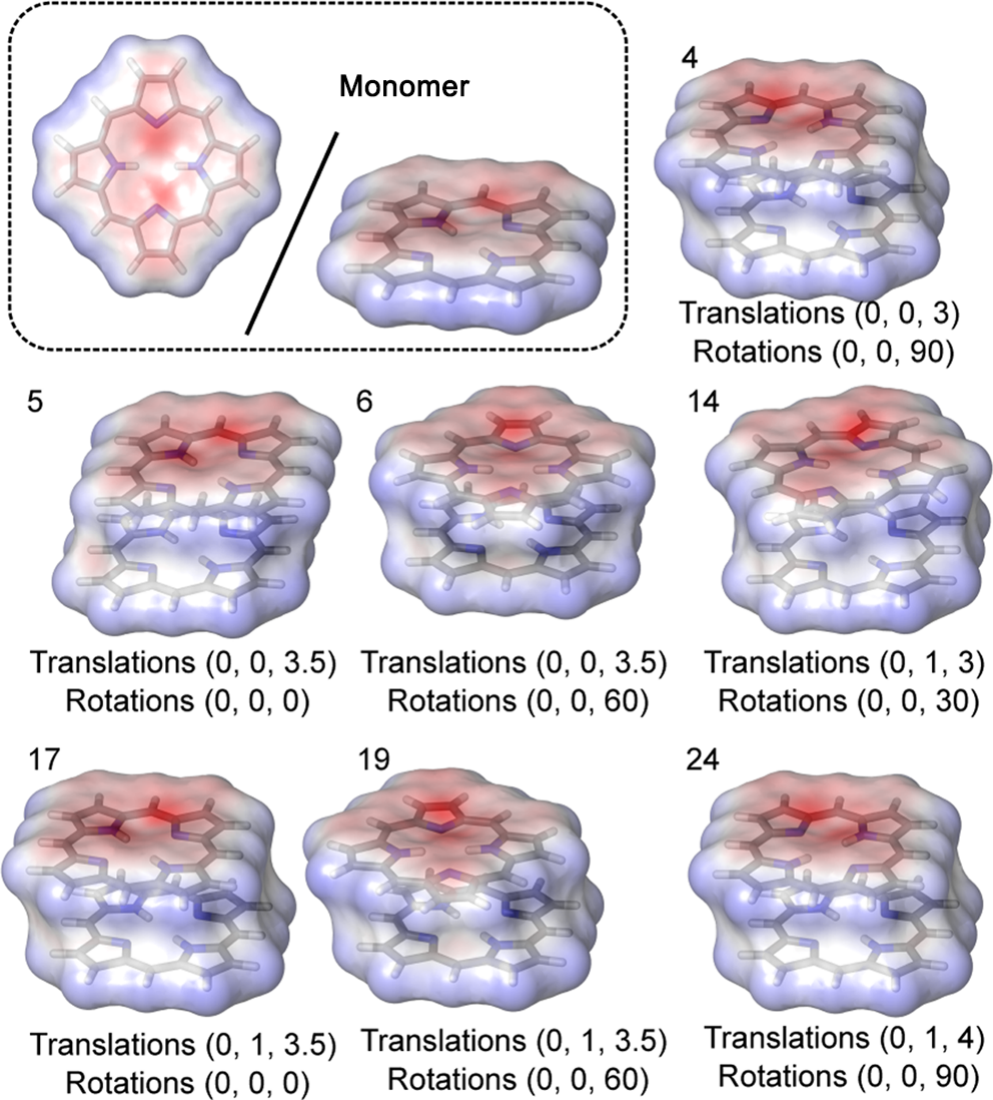
Comparison of electrostatic potential maps for various dimers calculated with CAM-B3LYP/6-311++g/vacuum.

Furthermore, the electrostatic potential maps (EPMs) were calculated for the optimized and energetically favorable structures (CAM-B3LYP/6-311++g/vacuum) on the solvent-accessible surfaces.^31,33,42^ The EPMs for the seven diverse minima structures obtained using the *n*RMSD = 0.015 Å threshold are presented in Figure 8, together with the EPM of the monomer. Notably, the regions of high negative charge associated with the nitrogen atom in the monomer exhibit local variations within the dimer structures. Naturally, the negative charge centers repel each other, so that molecules get either rotated when aligned directly on top of each other (i.e., the “sandwich” structure) or shifted.

### Discussion of Grid Sizes

The three grids used within this work served different purposes. While the “default” grid was used for the global structure navigation and local minima search, the “fine” grid was involved to check whether additional minima can be identified, which have been missed using the default grid. The “sandwich” grid was discussed in the previous section.

**Figure 9:**
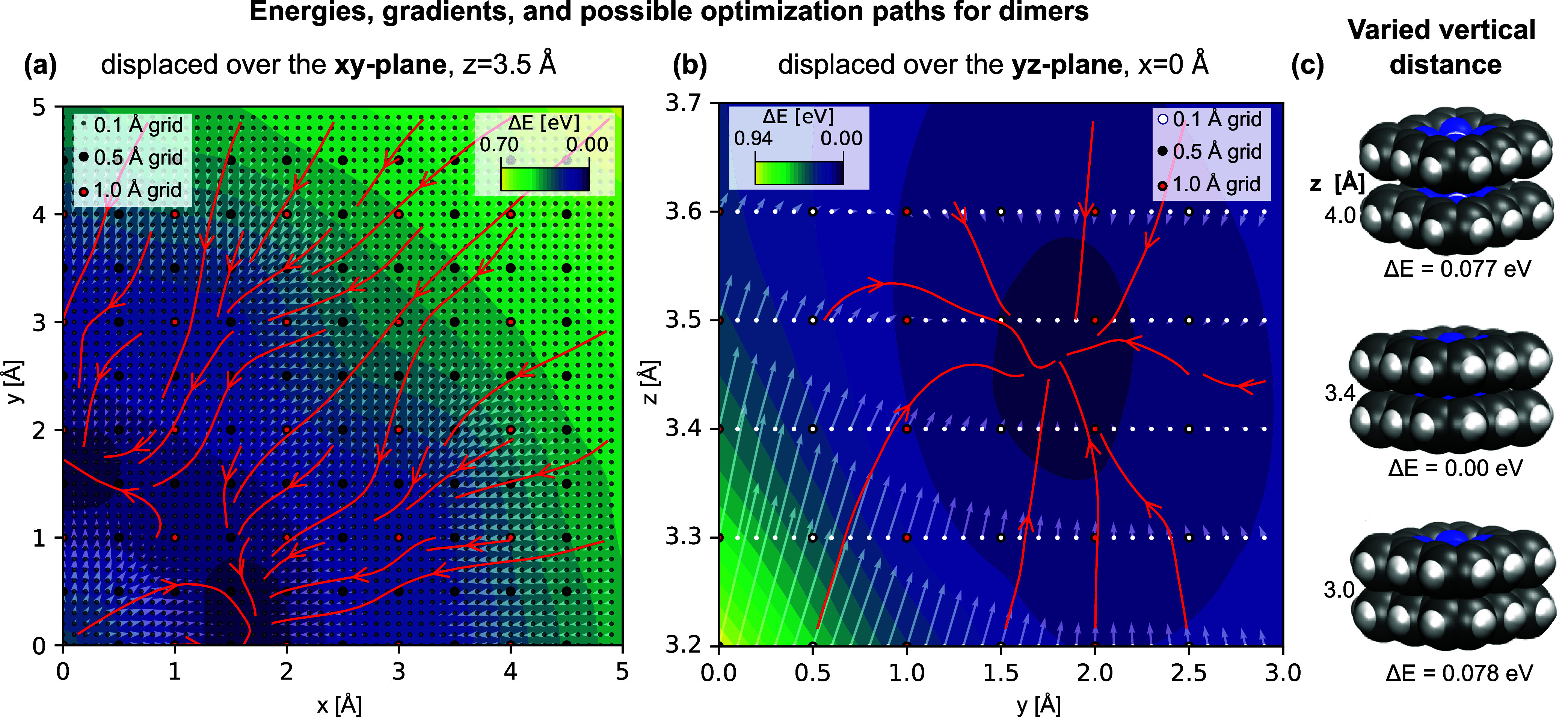
Energy contour maps (B3LYP/ahlrichs_pvdz/COSMO) for dimers where the second porphyrin is translated, but not rotated. The gray arrows depict the negative energy gradients for each “fine” grid point; The lengths of the vectors correlate with the magnitude of the gradient. Red arrows show the steepest descent energy paths starting from the points of the “1 Å grid”. Panel (a): Horizontal displacement over *x*- and *y*-axes (z = 3.5 Å). Panel (b): Vertical slice at *x* = 0 Å. Panel (c): Dimer structures for translations over *z* axis using the representation as van der Waals spheres to illustrate the vertical distance between the molecules.

Figure 9a exemplarily shows the energy landscape in the *xy*-plane for *z* = 3.5 Å and 0° rotation of the second molecule as resulting for the fine grid. From this map it becomes obvious, that steps of 1 Å are sufficient to generate starting points from which optimizers will develop the dimer geometries to end up in any all of the local minima present in the map, as visualized by the red-colored steepest descent paths in Figure 9. Moreover, even with steps of 2 Å both local minima dimers are identified for all cases studied (see Figure 9, and Figure S4 in the SI) if the z-distance matches approximately the van der Waals or π-stacking distance of ≈3.5 Å (cf. Figure 9b,c, and Figure S2a and S2b in the SI).

The density and extensions of the grids depend strongly on the respective molecule geometries. In order to study the scalability of the method, calculations on urea dimers and benzene dimers and trimers were performed and have proved to find the literature-known aggregate structures^6,14,43^ (details in Section 6 of the SI).

### Discussion of Functionals and Basis Sets

To study how much the density functional influences the results discussed above, we repeated the dimer search (focusing on the single-point calculations) based on the default grid for the range-corrected hybrid functional CAM-B3LYP and additionally varied the environment (vacuum versus perfect conductor model). Because numerous single-point calculations and many geometry optimizations are involved in our dimer search, the size of the basis set is important, as smaller basis sets result in faster calculations. To investigate whether the small basis set lanl2dz_ecp yields similar results as the before-employed basis set ahlrichs_pvdz, the dimer search was conducted for both basis sets in combinations with variations of the density functional and the environment.

**Figure 10:**
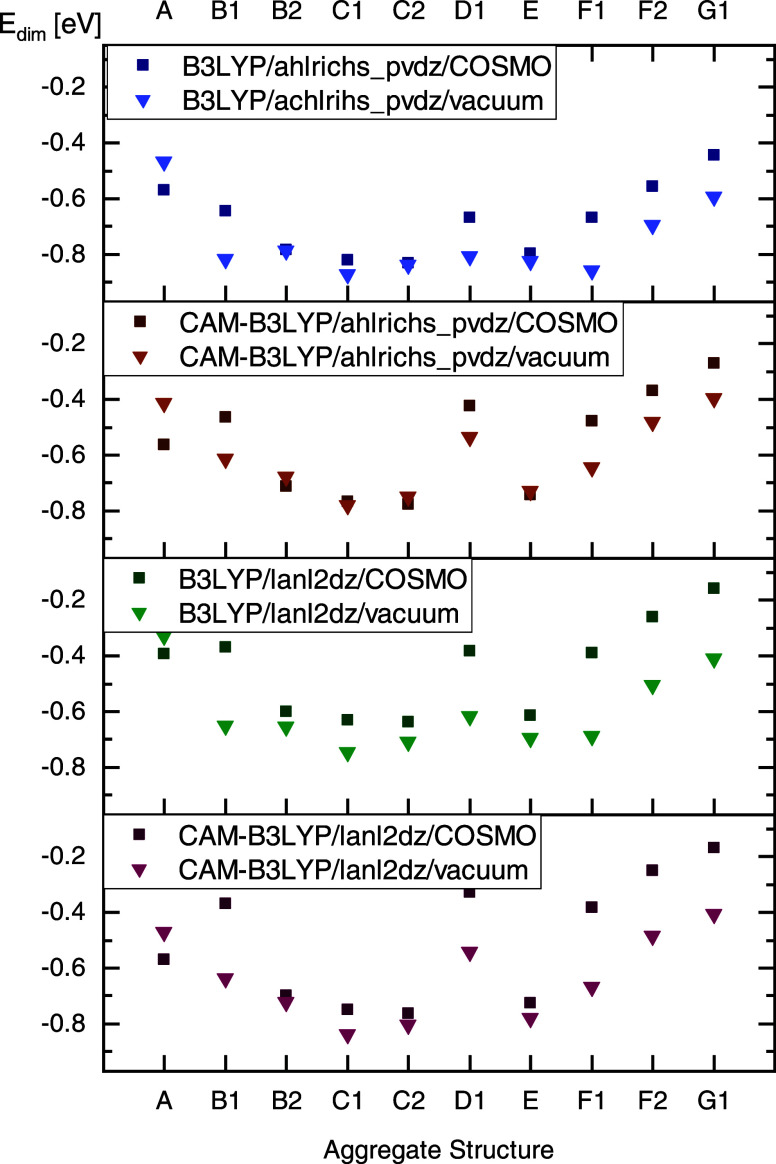
Comparison of single-point energies of the energetically most favorable dimers of the families discussed above for varied quantum chemical parameters. Vacuum and COSMO are both considered.

As shown in Figure 10, these variations in density functional, basis set, and environment do not alter the positions of local minima within the grids as calculated with single-point calculation, so that the quantitative pictures of Figures 4 and 5 are retained as seen in Figure S4 of the SI. Also, the influence of the functional and basis set on the relative energies among the dimers is small, as shown in Figure 10. The energy relation obtained with the ahlrichs_pvdz and lanl2dz basis sets are consistent, although lanl2dz is significantly smaller (resulting in faster quantum chemical calculations) than ahlrichs_pvdz. The only notable difference in energetic hierarchy is observed for dimer A for the conductor environment and the CAM-B3LYP functional, which yield significant stabilizations of the rotated “sandwich” structure A, thus ranging among the energetically rather favorable dimers.

The consideration of vacuum conditions or a polarizable environment leads to a changed energetic ranking of the obtained dimers. However, as mentioned above, the choice of density functional or basis set has only a minimal effect on the energetic order of the dimers investigated in this study. Therefore, an aggregate search that includes potential stabilization effects of surrounding molecules - using the COSMO model - proves to be ideal for material development. Moreover, even a small basis set may be sufficient for the initial identification of relevant aggregate geometries.

### Extension to Larger Aggregates

**Figure 11:**
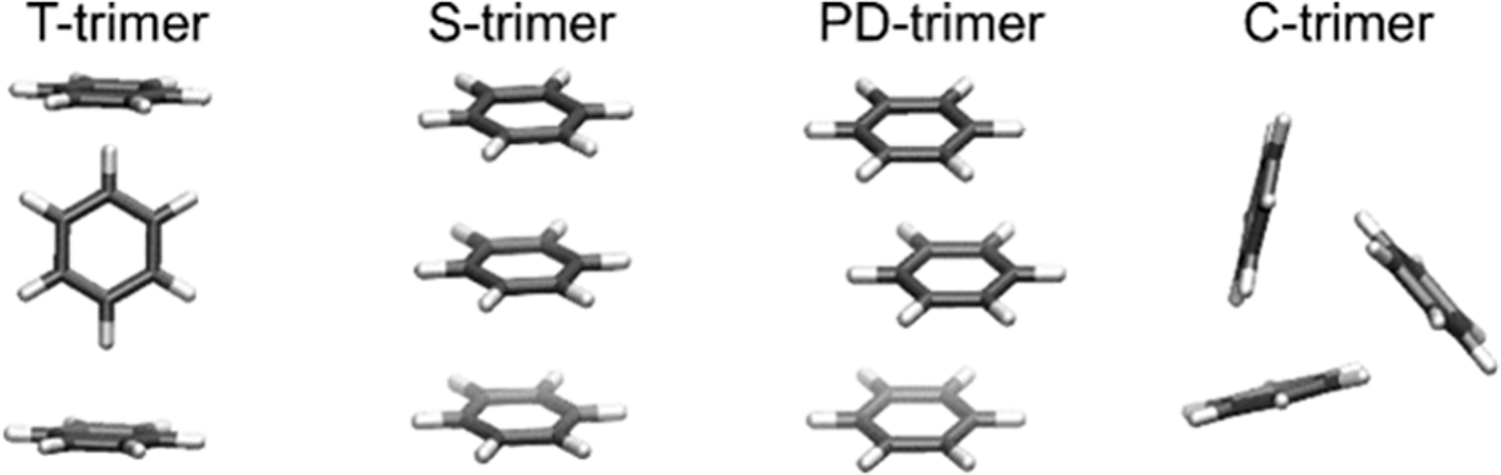
Benzene trimers found via our search algorithm, which correspond to those reported in literature.

Since the approach used so far is systematic, it can easily be used to calculate higher aggregates such as trimers. These are particularly well-known in the literature for benzene^44^ and are therefore predestined as an additional reference system. Therefore, first energetically favorable and geometrically diverse dimer structures are determined, thereby following the procedure introduced for porphyrin and described in Section 6.3 of the Supporting Information. Starting from the resulting set of dimers, they are translated to the different lattice points around a monomer in order to obtain trimer structures. According to the results of the porphyrin dimer study described above, we chose a translation lattice with a step size of 1.0 Å in all directions and an extension in the *z*-direction from ±4 to ±9 Å and in the *x*- and *y*-directions from 0 to6 Å, respectively. The optimization (B3LYP/ahlrichs_pvdz/COSMO) of the minima found by single-point calculations (B3LYP/lanl2dz/COSMO) and their selection by hierarchical clustering and energy evaluation yields all trimer structures known from the literature, C (cyclic), T (T-shaped), S (stacked), PD (parallel-displaced), as shown in Figure 11.

## Conclusions

The rapid ab initio prediction of potential aggregate structures is crucial for the advancement of organic semiconducting materials. The optical and electronic properties, and thus the functionality, of these materials are strongly influenced by the supramolecular geometry of the dyes that compose them. This includes the structure of the dye aggregates, which plays a key role in determining their performance characteristics. While previous systematic searches used very dense grids of dimer geometry variations and therefore required fast force field calculations to analyze the resulting billions of dimer geometries, our current research shows that translational grids with a spacing of 2 Å and rotational variations of 30° are sufficient to generate starting structures from which classical geometry optimizers optimize dimer geometries to all relevant energy minima. On such coarse grids, these geometry optimizations can be performed with ab initio methods due to their comparatively small number, and DFT has been shown to be suitable even for very small basis sets. We have also shown that hierarchical clustering, successfully used in OpenBabel-based similarity analysis, is ideally suited to partition the resulting dimers into structural families, significantly reducing the effort required for further refinement. Thus, our work demonstrates that systematic and ab initio prediction of molecular dimers, especially those containing extended π-electron systems, is already possible today and paves the way for fast and reliable routine prediction of molecular aggregate structures. The application of our systematic approach to the prediction of larger aggregate structures is methodologically straightforward, as shown by the example of benzene trimers.^45^

## Supporting Information Available

Details on translational and rotational grid analysis, including showing the translational symmetry of energy landscapes; analysis of the gradients of the energy field, obtained with the “fine” orthorhombic grid at different *z* values; local minima points for different quantum chemical parameters, correspondence of the dimers with their energies and Boltzmann distribution; details on identification of the “sandwich” aggregate by spanning rotations and translations separately; comparison of clustering algorithms of the aggregate geometries; further quantum chemical calculations, including correction of basis set superposition error (BSSE) and employing different quantum chemical parameters for determining local minima over *xy*-plane; comparison with aggregate generation via the CREST algorithm; Showing the scaling of grid sizes for urea dimer search, benzene dimer search, benzene trimer search (PDF)
