# Supplementary material for: Catching the π-Stacks: Prediction of Aggregate Structures of Porphyrin
Source: J Phys Chem A. 2024 Nov 9;128(46):9917–26. doi: 10.1021/acs.jpca.4c05969 (PMC11586908; doi:10.1021/acs.jpca.4c05969)
Supplement: Supplementary file 1 — jp4c05969_si_001.pdf [file jp4c05969_si_001.pdf]

# - Supporting information -

Catching the π-Stacks: Prediction of Aggregate Structures of Porphyrin

Anna Elmanova[1,2,3], Burkhard O. Jahn[3], Martin Presselt[1,2,3,4*]

[1] Institute of Physical Chemistry, Friedrich Schiller University Jena, Helmholtzweg 4, 07743 Jena, Germany

[2] Leibniz Institute of Photonic Technology (IPHT), Albert-Einstein-Str. 9, 07745 Jena, Germany

[3] SciClus GmbH&Co. KG, Moritz-von-Rohr-Str. 1a, 07745 Jena, Germany

[4] Center for Energy and Environmental Chemistry Jena (CEEC Jena) Friedrich Schiller University Jena, Philosophenweg 7a, 07743 Jena, Germany

*Corresponding author: martin.presselt@leibniz-ipht.de

Contents

1. Analysis of translational and rotational grids 2

1.1. Translational symmetry 2

1.2. Local minima of the energy landscapes 2

1.3. Rotational symmetry of local minima points 4

2. Identifying the “Sandwich” aggregate 6

3. Clustering of the aggregate geometries 7

4. Further quantum chemical calculations 8

4.1. Basis set superposition error (BSSE) 8

4.2. Different quantum chemical parameters when determining local minima 10

5. Aggregate generation via the CREST algorithm 12

6. Scaling of the grid sizes 12

6.1. Urea dimer search 12

6.2. Benzene dimer search 13

6.3. Benzene trimer search 13

1. Analysis of translational and rotational grids

1.1. Translational symmetry

Porphin is highly symmetric over x and y axes for the non-rotated (“sandwich”) structure. This can be easily shown by the heatmaps for energy distributions over x and z and over y and z axes (Figure S1). The picture for translation over x and y with the same slicing over other left axis gives identical energy distribution.

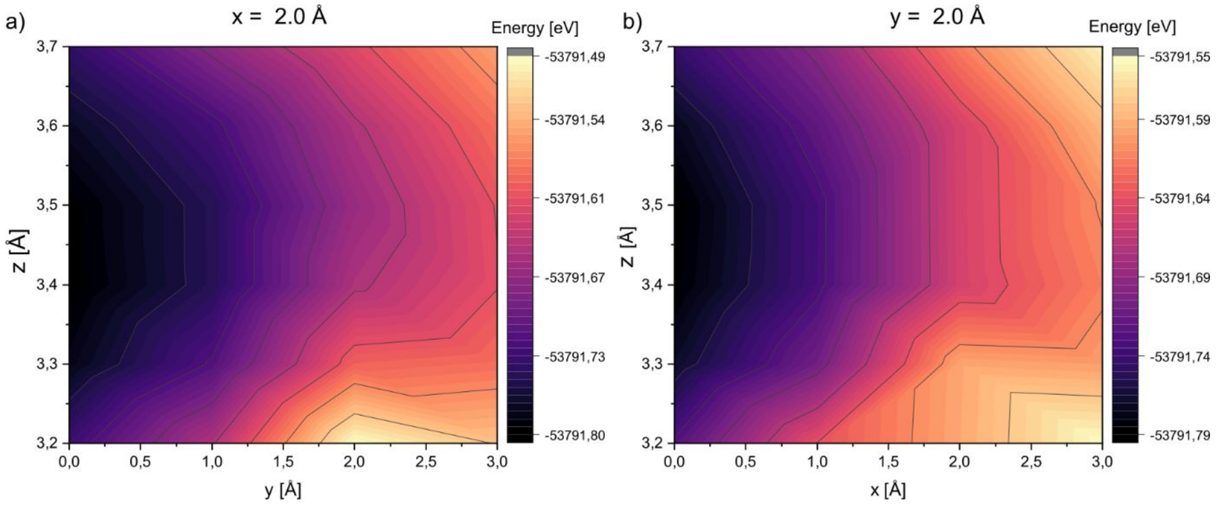

Figure S1. Heatmaps of the energy distributions over different axes for fine z grid. Panel (a): For constant x values. Panel (b): For constant y values. The second porphin is not rotated against the initial one.

1.2. Local minima of the energy landscapes

The local minima for energy landscapes in the present paper were revealed by analysing the gradients of the energy field, obtained with the “fine” orthorhombic grid. In Figure S2 and Figure S3 below the negative gradient directions in each fine grid point are shown as white arrows, while the red arrows show potential optimization trajectories following the steepest slopes when starting from the 1 Å-spaced grid. These plots reveal that the that a 1 Å-spaced grid is sufficient to reach all local minima.

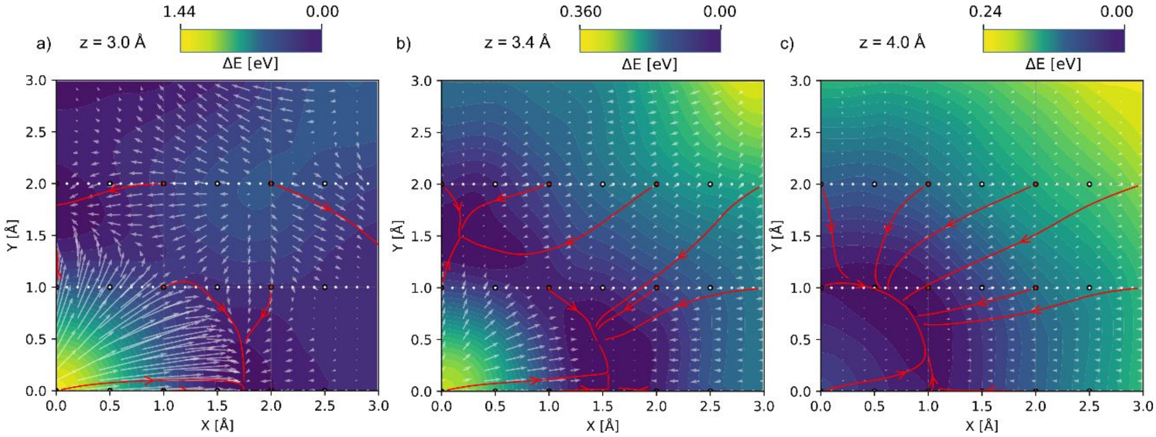

Figure S2. Heatmaps of the energy distributions over x and y axis for constant translations over z (rotation 0 degrees).

In Figure S2 the energy landscapes for different displacements in z-direction are shown. It is noticeable that for both z=3.0 Å- and 3.4 Å-slices two energy minima are obtained, which are merged into a single one in the z=4.0 Å-slice. Figure S3 shows energy landscape for constant displacement in z-direction, but different rotations of the second porphyrin, resulting in a symmetric picture for non-rotated aggregate and a less symmetric energy landscape for 30° rotation.

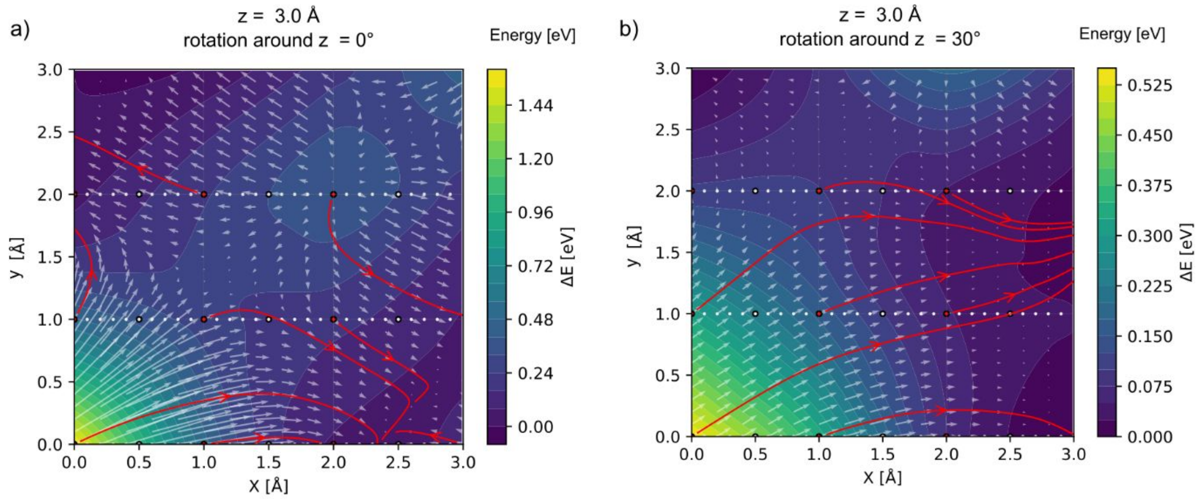

Figure S3. Heatmaps of the energy distributions over x and y axes with constant z levels. Panel (a): For rotation around z axis 0 degrees. Panel (b): Same for 30 degrees. Points denote grid of 0.5 Angstrom. The flow lines depicted with arrows for both positive and negative gradients.

1.3. Rotational symmetry of local minima points

The different local minima points, obtained via navigation over the energy landscapes for different quantum chemical parameters are presented in Figure S4.

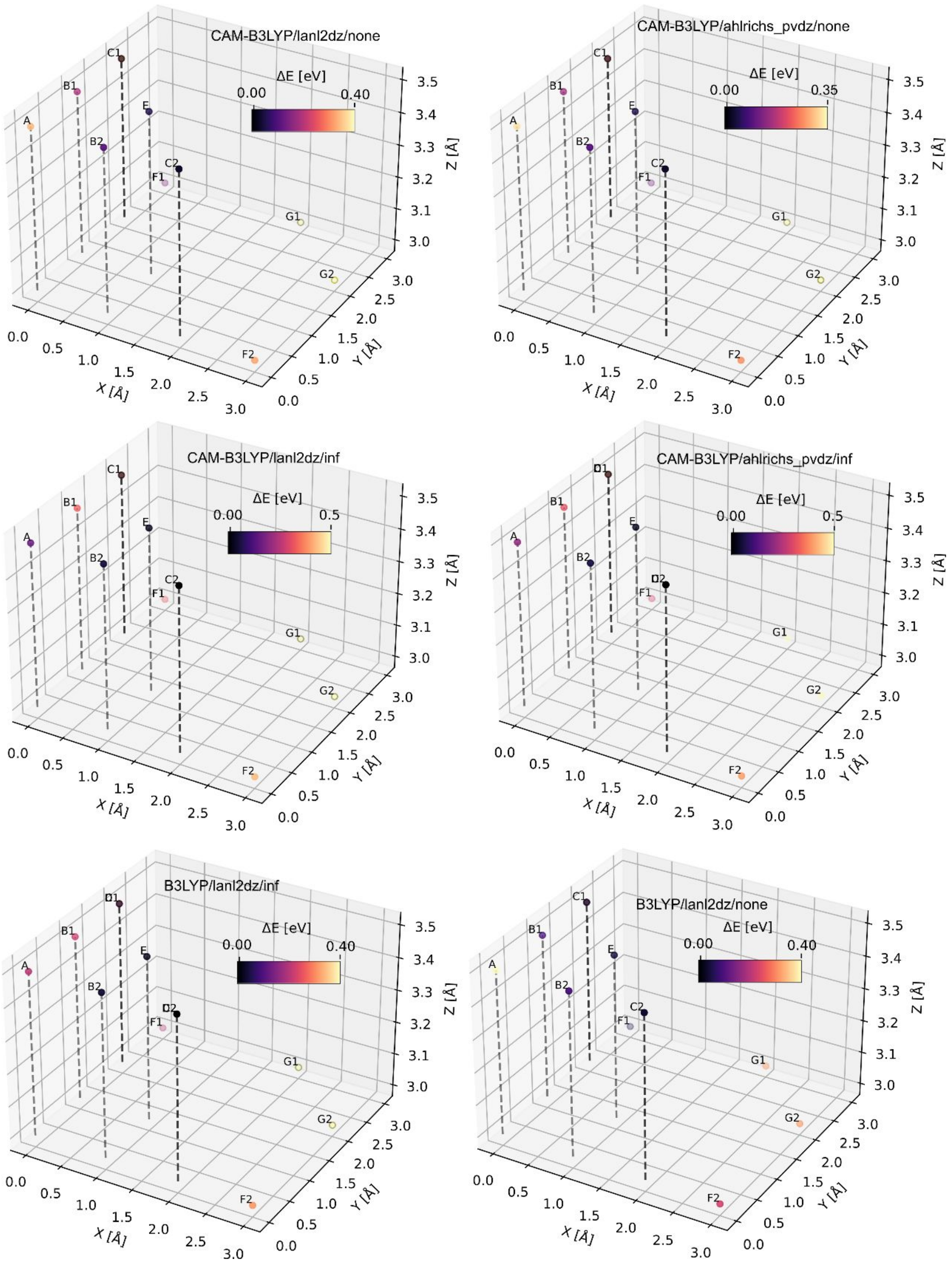

Figure S4. Different energetical local minima structures, found by direct comparison, for various QC parameters.

Table S1 provides supplementary details for Figure 5 in the main text, specifically focusing on the rotations identified for various translations. It's essential to note that the rotations listed are only those that converged during the optimization process. The

search encompassed rotations around the z-axis spanning from 0 to 360°. Notably, angles of 30, 60, 90, and 0° were duplicated due to inherent symmetry, resulting in the identification of multiple structures in each repetition.

Table S1. Rotations over different translational local minima found while global energy navigation with energy values for B3LYP/ahlrichs_pvdz/COSMO.

x [Å]	y [Å]	z [Å]	Structure	Energy [eV]	Rotation [°]	ΔE [eV]	ΔE [kJ/mol]	P(E) [%]
0,00	0,00	3,50	A	-53824,29	60,00	0,201	19,429	9,3643E-03
0,00	1,00	3,50	B1	-53824,47	60,00	0,017	1,672	1,3710E+01
1,00	0,00	3,50	B2	-53824,47	60,00	0,017	1,670	1,3721E+01
0,00	2,00	3,50	C1	-53824,49	90,00	0,000	0,000	2,7235E+01
2,00	0,00	3,50	C2	-53824,49	90,00	0,002	0,229	2,4787E+01
0,00	2,00	3,00	D1	-53824,32	90,00	0,163	15,699	4,3299E-02
2,00	0,00	3,00	D2	-53824,33	90,00	0,162	15,668	4,3851E-02
1,00	1,00	3,50	E	-53824,48	60,00	0,009	0,820	1,9450E+01
0,00	3,00	3,00	F1	-53824,20	60,00	0,286	27,564	3,3208E-04
3,00	0,00	3,00	F2	-53824,23	60,00	0,260	25,084	9,1935E-04
2,00	3,00	3,00	G1	-53824,20	60,00	0,286	27,641	3,2172E-04
3,00	2,00	3,00	G2	-53824,20	60,00	0,288	27,825	2,9840E-04

Table S2. Correspondence of the dimers with their energies and Boltzmann distribution probabilities from the optimized aggregate analysis with probabilities calculated for CAM-B3LYP/ahlrichs_pvdz/COSMO.

NoConf	E[eV]	ΔE[eV]	P(E) [%]	E_{dim} [eV]					
					18	-53792,00000	0,0300	2,177691	-0,95468
1	-53792,03000	0,0000	7,000053	-0,98468	19	-53791,98000	0,0500	0,99983	-0,93468
2	-53791,97000	0,0600	0,677472	-0,92468	20	-53792,03000	0,0000	7,000053	-0,98468
3	-53791,96000	0,0700	0,459046	-0,91468	21	-53792,02000	0,0100	4,743144	-0,97468
4	-53791,97000	0,0600	0,677472	-0,92468	22	-53792,00000	0,0300	2,177691	-0,95468
5	-53792,02000	0,0100	4,743144	-0,97468	23	-53791,99000	0,0400	1,475575	-0,94468
6	-53791,97000	0,0600	0,677472	-0,92468	24	-53792,03000	0,0000	7,000053	-0,98468
7	-53791,97000	0,0600	0,677472	-0,92468	25	-53792,02000	0,0100	4,743144	-0,97468
8	-53791,83000	0,2000	0,002913	-0,78468	26	-53792,00000	0,0300	2,177691	-0,95468
9	-53791,82000	0,2100	0,001974	-0,77468	27	-53791,99000	0,0400	1,475575	-0,94468
10	-53791,97000	0,0600	0,677472	-0,92468	28	-53792,03000	0,0000	7,000053	-0,98468
11	-53791,97000	0,0600	0,677472	-0,92468	29	-53792,02000	0,0100	4,743144	-0,97468
12	-53791,83000	0,2000	0,002913	-0,78468	30	-53792,00000	0,0300	2,177691	-0,95468
13	-53792,02000	0,0100	4,743144	-0,97468	31	-53791,99000	0,0400	1,475575	-0,94468
14	-53791,99000	0,0400	1,475575	-0,94468	32	-53792,03000	0,0000	7,000053	-0,98468
15	-53791,98000	0,0500	0,99983	-0,93468	33	-53792,02000	0,0100	4,743144	-0,97468
16	-53792,03000	0,0000	7,000053	-0,98468	34	-53792,00000	0,0300	2,177691	-0,95468
17	-53792,02000	0,0100	4,743144	-0,97468	35	-53791,99000	0,0400	1,475575	-0,94468

2. Identifying the "Sandwich" aggregate

For a detailed characterization of energetically favorable π-stacks, we systematically explored different rotations within the dimers. Figure S5a shows the local energy minimum for displacement over the z-axis (without rotation), while Figure S5b shows the minima observed for rotation around the z-axis. The energy minima for displacements along the x- and z-axes are shown in Figure S5c. For the "sandwich" stacking, we fixed the translation at (0, 0) along the x- and y-axes and found that the unoptimized "sandwich" structure rotated by 60° is optimized to a 45° rotation, which is consistent with the one-point calculations. This non-rotated structure and the dimer with the porphyrin rotated by 90° represent local maxima, not minima. This leads to rotations or translations during geometry optimization, as shown in Figure S5d.

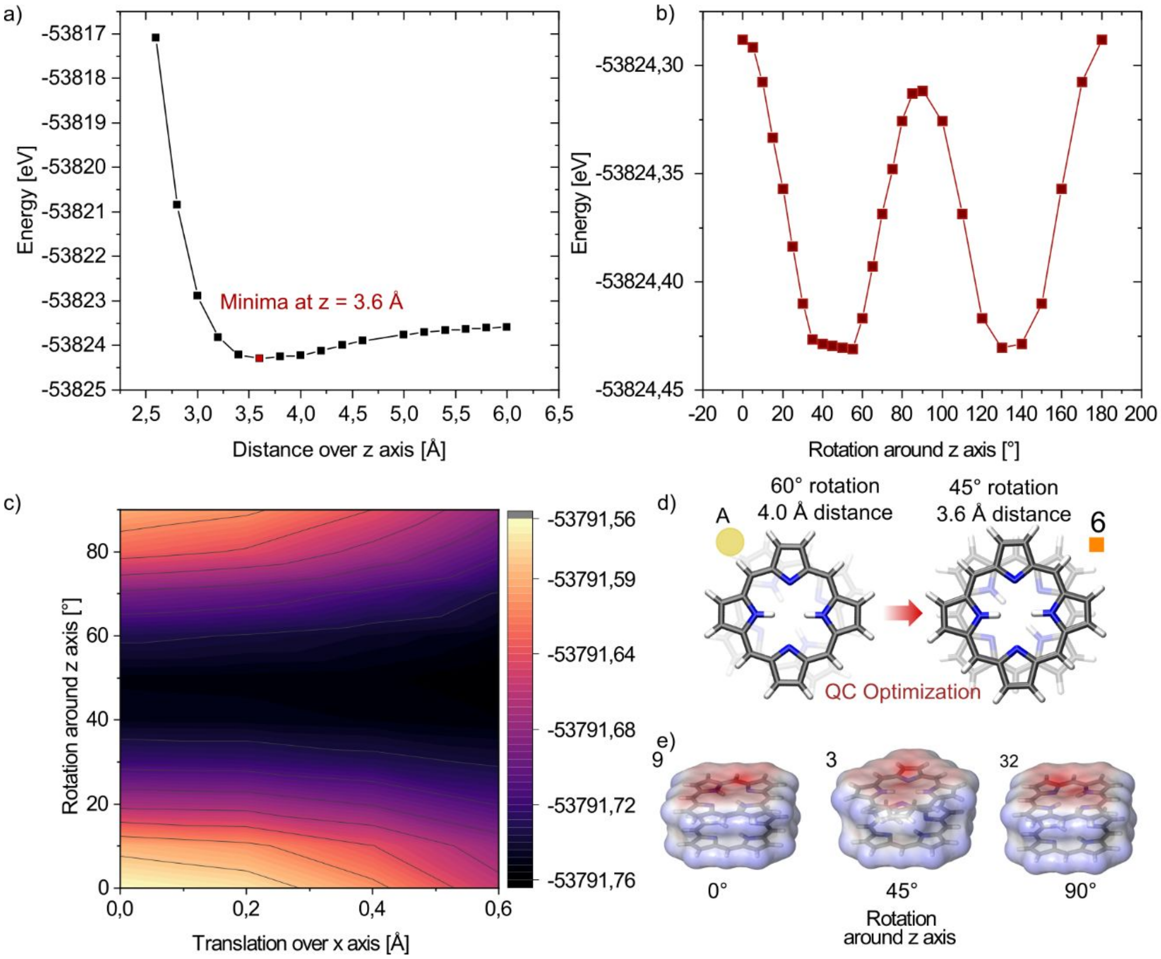

Figure S5. Panel (a): Single point energies for non-rotated structure, translated only over z axis. Red point denotes the minima point. Panel (b): Energies for the 3.6 Å distance over z axis displaced "sandwich" structures of different rotations. Panel (c): Displacements over x and rotations of the structure at z equal to 3.6 Å. Panel (d): Local minima structure found within the local minima single-point energy search and the optimized version of the same structure (calculations with B3LYP and ahlrichs-pvdz). Panel (e): Determination of the energetically favorable rotation for the "sandwich" structure. Calculations of energies done for B3LYP/ahlrichs_pvdz/COSMO, the SPMs calculated with CAM-B3LYP/6-311++g/vacuum.

3. Clustering of the aggregate geometries

Different clustering algorithms are employed in aggregate and conformation analysis. As far as both problems (distinguishing aggregates and conformations) are about comparing molecular geometries with energy ranking, we can refer to both in terms of comparing different clustering methodologies. In the work by Kim et al.[1] authors compared different clustering algorithms, using conformer sets with RMSD metric. Authors highlight the question of complications in finding good determination of a favorable cluster. The k-means clustering was found to be disadvantageous due to its impossibility to determine the global optimum and the need to specify the number of clusters, k, which in general can affect the result of clustering significantly. The authors suggest using an improved k-means algorithm with automated search of k which would minimize the mean of the squared distance of the overlap within clusters and to maximize the mean of the squared distance of the overlap between the clusters. The authors also used hierarchical clustering in their searches, namely the hierarchical clustering with the dynamical threshold, which can be applied for complex trees, for which the static method (with the same threshold) is not always applicable. The tree was constructed based on the Ward's minimum variance distance. The authors claim that dynamic tree-based clustering has shown itself as the most desirable clustering method presenting a consistent reduction of the data, the small size of a sample, and a high coverage of conformational space. This study has shown that dynamic hierarchical clustering is highly superior in comparison with improved k-means clustering. Of course, direct comparison with our methos would be incorrect, as we apply static hierarchical clustering in the present work. Another work, by Shao et al.[2] studied the question of different clustering algorithms for clustering molecular dynamics trajectories, which is again a similar problem. This work has shown weaknesses of both k-means and hierarchical clustering, implying that the hierarchical clustering suffers from the uniform/linear cutting, and the k-means tends to create clusters of uniform sizes, while centroid-linkage, centripetal, and Bayesian algorithms were able to create clusters with distinct shapes and sizes. Still, in our paper we can distinguish between highly different sizes of clusters (see Figure 6 of the main text), i.e. clusters consisting of single conformation and those comprising many conformers, even when using low thresholds (c24-c20 family with lower threshold shows six conformations).

To evaluate our hierarchical clustering, e.g. at the Figure 6 of the main text, we performed an k-means clustering according the following procedure:

1. The energies and RMSD values between the different dimers are represented by a symmetric matrix. The diagonal elements of the RMSD matrix, which should prove identity of a dimer with itself, are usually not exactly zero usually around 10^{-10} Å and lower and are, hence, set to zero.
2. The program finds the optimal number of clusters (k) using the silhouette score. The silhouette score measures how similar an object is to its own cluster (cohesion) compared to other clusters (separation). The code iterates through different values of k and selects the value that maximizes the silhouette score.
3. Then k-means clustering is performed with the optimal number of clusters (k) obtained in the previous step.
4. The code at the end creates a table with the clustering information. The first row shows the dimer name, and the second row contains their corresponding cluster numbers. An example is presented in Table 3.

The result of k-means clustering is identical to that of hierarchical clustering if an RMSD threshold of 0.02 Å is used for the latter.

Table 3. Results of the k-means clustering with dynamic k value for the structures from Figure 6 of the main text, compared with the family grouping according to hierarchical clustering, shown in the main text. The order of families was changed to facilitate comparison with k-means clustering, but the order of dimers inside each family is shown according to original hierarchical clustering (and to the main text figure).

(a) Dimers, grouped to families according k-means clustering with dynamic *k* value														
Dimer No.	33	17	21	13	29	25	35	23	27	31				
Family	0	0	0	0	0	0	1	1	1	1				
Dimer No.	2	6	10	11	7	3	32	16	20	24	28	36		
Family	2	2	2	2	2	2	3	3	3	3	3	3		
Dimer No.	34	26	14	22	18	30	4	9	5	1	12	8	15	19
Family	4	4	4	4	4	4	5	6	6	6	7	7	8	8

(b) Dimers, grouped to families according to hierarchical clustering with threshold 0.02 Å														
Dimer No.	29	25	33	21	13	17	35	31	27	23				
Family	6	6	6	6	6	6	0	0	0	0				
Dimer No.	10	6	11	7	2	3	20	16	36	32	28	24		
Family	2	2	2	2	2	2	3	3	3	3	3	3		
Dimer No.	34	30	26	22	18	14	4	1	5	9	12	8	19	15
Family	7	7	7	7	7	7	8	5	5	5	4	4	1	1

4. Further quantum chemical calculations

4.1. Basis set superposition error (BSSE)

To check the magnitude of BSSE and its potential influence on our results and conclusions, we applied the Boys-Bernardi counterpoise correction, calculated using the following formula[3]:

$$E_{AB}^{counterpoise} = E_{AB}(H_{AB},R_A,R_B;A \cup B) - E_A(H_A,R_A;A \cup B) - E_B(H_B,R_B;A \cup B),$$

where the first arguments in the parentheses are the Hamiltonian and coordinates used and the second arguments (after the semicolon) are the basis sets used. Therefore, the molecular orbitals include the basis set functions for both porphyrins, but not the electrons and nuclei of the second.

The BSSE corrected dimerization energies were calculated as follows:

$$E_{BSSE-corrected} = E_{dimer} - E_{AB,\,A\;ghost} - E_{AB,\,B\;ghost},$$

In this procedure, E_{dimer} represents the total energy of the dimer, while the other two components are calculated by treating the atoms of one molecule in the dimer as so-called ghost atoms. The BSSE-corrected and not-corrected energies as well as their difference are shown in Figure S6 (B3LYP/ahlrichs_pVDZ/vacuum). If the heatmaps of the BSSE-corrected and non-corrected energies automatically color-scaled (Figure S6b,d) they hardly differ. Therefore, also heatmaps using the same range of energies for the color-scaling are shown (Figure S6a,c). The difference between BSSE-corrected and non-corrected energies is also shown (Figure S6e,f). Notably, the differences observed are within the expected literature ranges[4]. The BSSE error did not affect the positions of local minima or maxima but did have a minor influence on the plateau regions. Consequently, the method used to identify local minima in the dimer searches remained robust and was not compromised by the presence of BSSE.

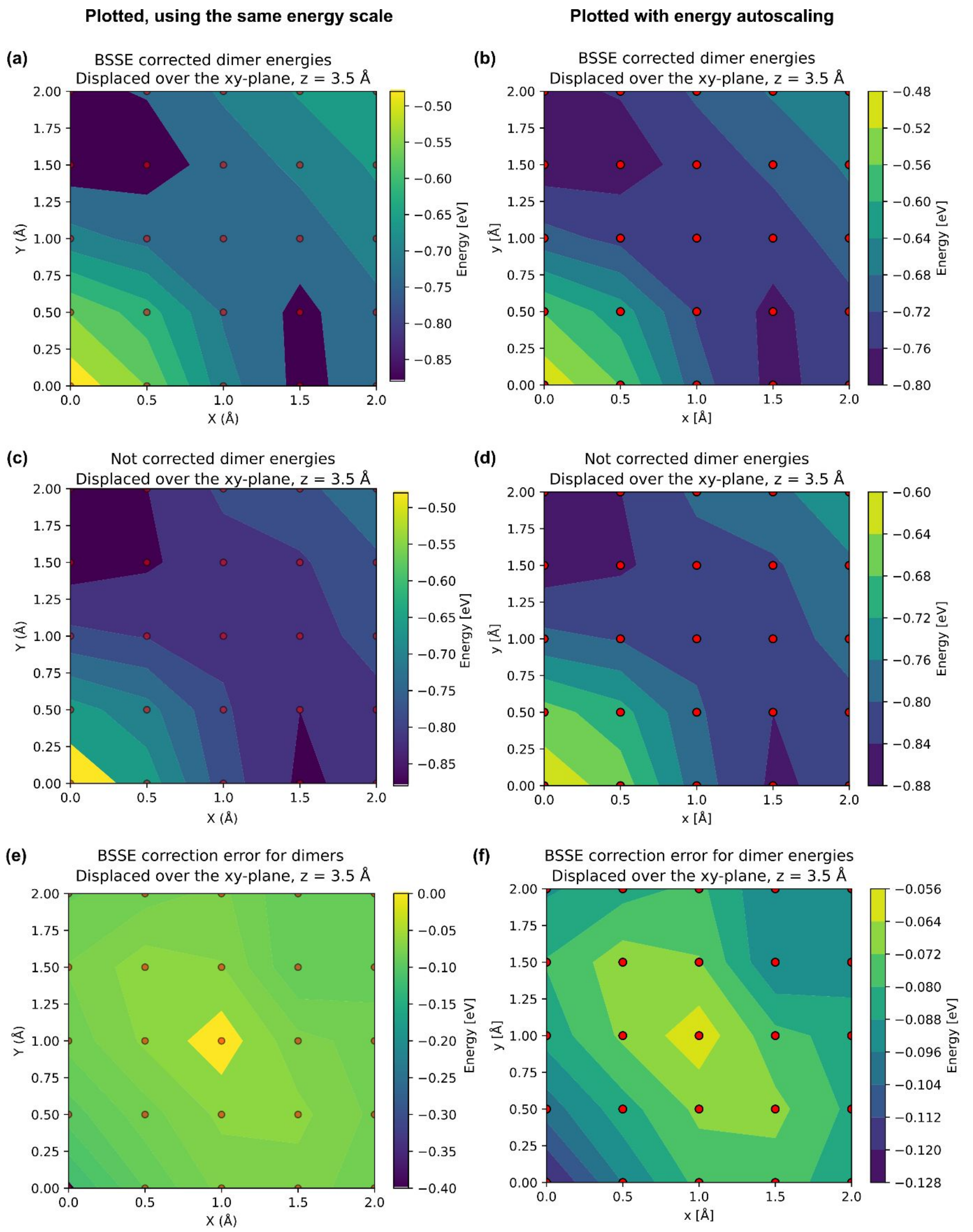

Figure S6. Energy landscapes for porphyrin dimers without rotation, displaced differently over the xy-plane with constant z = 3.5 Å level. In the left column the plots have the same energy scale for panels (a) and (c) and same, but offset for (e), while in the right column all the plots are auto-scaled. Red points denote the translations, at which dimers were generated and energetically evaluated. All calculations are done at the B3LYP/ahlrichs_pvdz/vacuum level. Panel (a): BSSE corrected dimer energies with fixed energy scale. Panel (b): Same as in panel a, but with auto-scaled color. Panel (c): Dimerization energy, calculated as in the rest of the paper. Panel (d): Same as panel c, but with auto-scaled color. Panel (e): Difference between energies, presented in panels (a) and (c) with the same energy scale. Panel (f): Same, but with own auto-scaled energy bar.

4.2. Different quantum chemical parameters when determining local minima

In addition to searching for local minima using the default grid settings with B3LYP/CAMB3LYP, ahlrichs_pVDZ/LANL2DZ, and COSMO/vacuum environments, local minima were also explored for porphyrin dimers using the REVPBE0 functional combined with the 6-311++G basis set in both COSMO and vacuum environments. These additional searches were conducted using a coarse grid. Rather than determining the local minima across the entire dimer space (like in 1.3 of the present SI), the minima were identified for a specific configuration of non-rotated dimers displaced over the xy-plane at a constant z = 3.5 Å, to facilitate a straightforward comparison of minima positions.

As depicted in Figure S7, this comparison reveals the differences between the REVPBE0 and B3LYP functionals when paired with the ahlrichs_pVDZ basis set, and when used both vacuum and COSMO environments. It also highlights the contrast between the 6-311++G and ahlrichs_pVDZ basis sets when applied with the REVPBE0 functional under the same environmental conditions. Although the dimerization energies varied in absolute values, they remained within the expected literature range[5, 6], and the overall qualitative trends were consistent across all parameter combinations. Notably, the distinction between plateau regions and local minima was slightly reduced for REVPBE0/6-311++G/COSMO, but remained pronounced in REVPBE0/6-311++G/vacuum, as illustrated in panels (c) and (d) of Figure S7.

In conclusion, while the specific energy values varied, the general behaviour of the porphyrin dimers remained consistent across different functional and basis set combinations. This consistency underscores the reliability of the used B3LYP/ahlrichs_pvdz/COSMO parameters, as employed in the main text, and the robustness of the observed trends, regardless of the environmental or computational settings. The slight differences in the plateau regions, particularly under vacuum conditions, highlight the subtle impact of the computational parameters on the energy landscape of these molecular systems.

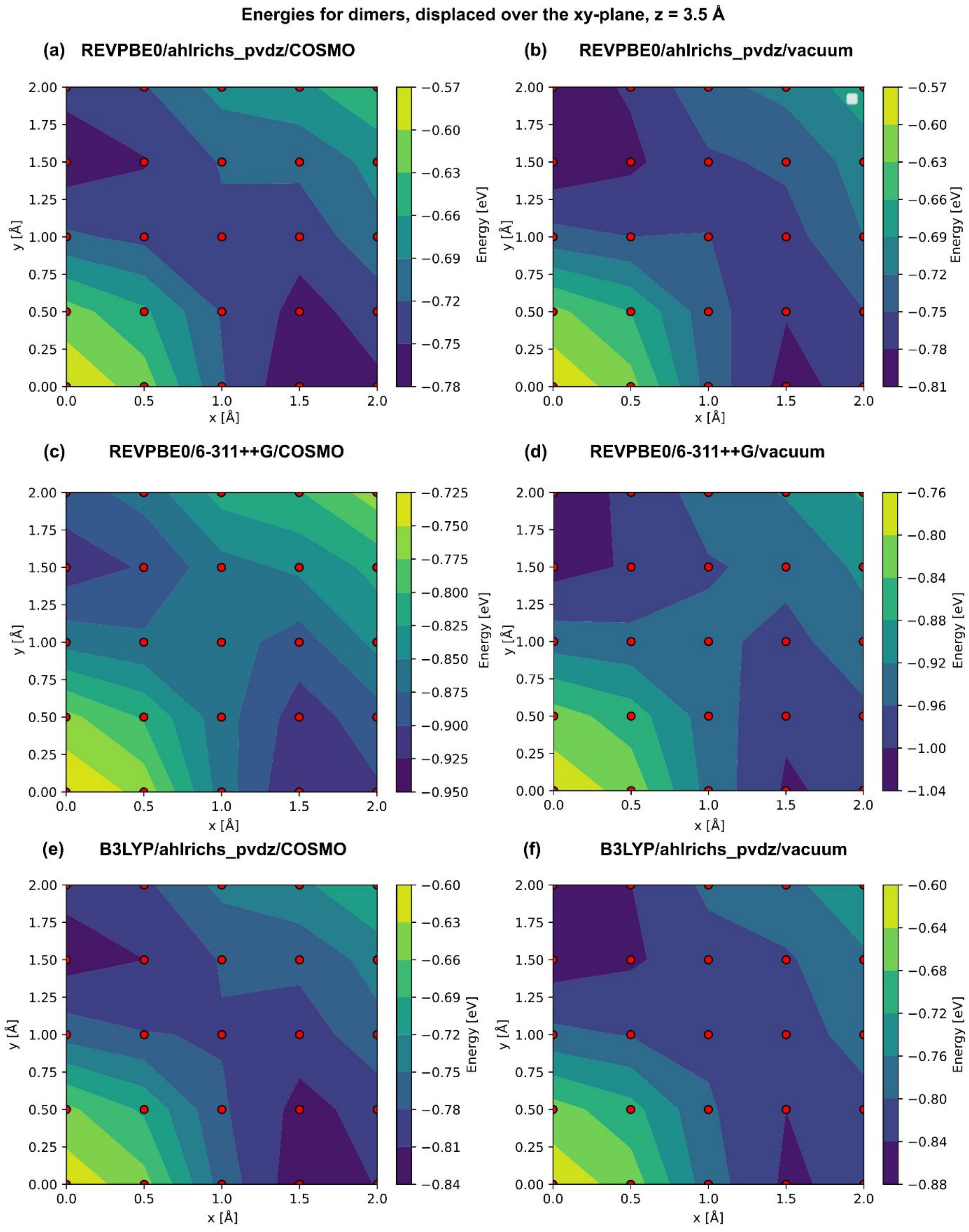

Figure S7. Energy landscapes for porphyrin dimers with no rotation, displaced differently over the xy-plane with constant z = 3.5 Ǻ level.

5. Aggregate generation via the CREST algorithm

In this chapter, we compare our systematic quantum chemical aggregate search with molecular dynamics methods, in particular the CREST algorithm (NCI mode).[7]

The CREST algorithm finds numerous sandwich dimers when the original input structure is a sandwich aggregate, but fails to find dimers that are significantly slip-stacked or rotated, i.e., which are geometrically quite distinct from the starting structure, if default parameters are used. CREST did not sample aggregates with a z-distances larger than 3.102 Å and not more distant in x- or y-direction than 1.52 Å. In contrast to the EnergyScan program, the CREST algorithm failed to determine T-shaped and highly x,y-displaced aggregates, cf. the local minimum aggregates with shifted up to 3.0 Å over x and y axes determined in this paper (see Fig. 5 of the main text). Still, the aggregates considered as rotamers cover greatly the rotational space around z axis. The aggregates were energy ranked by CREST and 23 structures were found as energetically favourable and diverse by CREST (see Figure S8). However, this search did not find the desired "9A" aggregate from Fig. 6 of the main text.

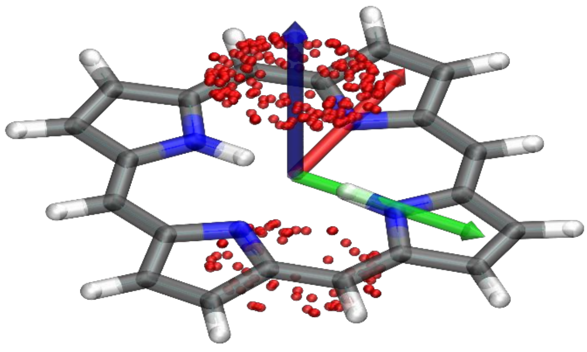

Figure S8. An image of a porphyrin monomer with the red spheres denoting positions of the second porphyrin in different dimers, generated with default -NCI mode of CREST software.

Consequently, our systematic DFT-energy calculations are fast and robust enough to be used for the primary energy ranking for systematic aggregate searches. Because they do not allow to miss several geometrical structures and do not depend on initial starting structures of the aggregates they provide a valuable route for aggregate structure predictions.

6. Scaling of the grid sizes

6.1. Urea dimer search

Urea dimers are extensively studied in the literature, making them an ideal test case for our aggregation algorithm[6, 8]. The literature suggests that the optimal distance between urea molecules in a dimer is around 2 Å. Based on this, we used z-axis displacements of 1.5, 2.0, 2.5, and 3.0 Å in our study. While various urea aggregates have been explored in previous research, our focus was on validating the procedure by examining only the parallel-displaced dimers and those rotated 180° around the x-axis. The grid used is seen in Figure S9. Single-point energy calculations for these urea dimers were performed using the REVPBE0/6-311++G/COSMO method. As expected, the known literature dimer configurations were successfully obtained using the corresponding grids and can be seen with their energies in Figure S10.

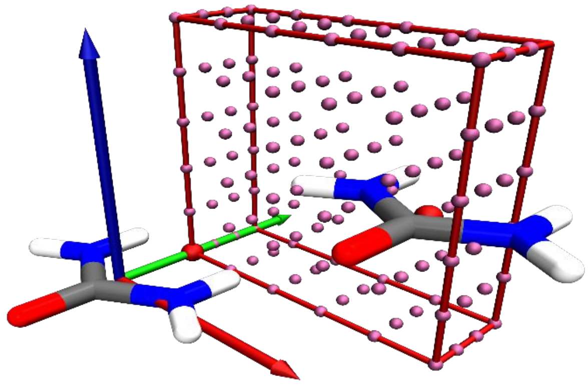

Figure S9. Translational grid for urea molecule dimer.

(a) Energies and spatial positions of local minima urea dimers

NoDimer	x [Å]	y [Å]	z [Å]	E [eV]	ΔE [eV]
1	0,0	-1,50	4,0	-12255,286	0,184
2	4,0	-2,50	2,0	-12255,268	0,202
3	4,0	-1,50	3,0	-12255,271	0,198
4	4,0	-3,00	2,0	-12255,274	0,195
5	5,0	-1,50	2,0	-12255,274	0,196
6	5,0	-3,00	1,0	-12255,268	0,201
7	0,00	-1,50	4,00	-12255,3	0,173
8	3,00	-3,00	3,00	-12255,27	0,197
9	4,00	-1,50	3,00	-12255,28	0,188
10	4,00	-3,00	0,00	-12255,47	0,000

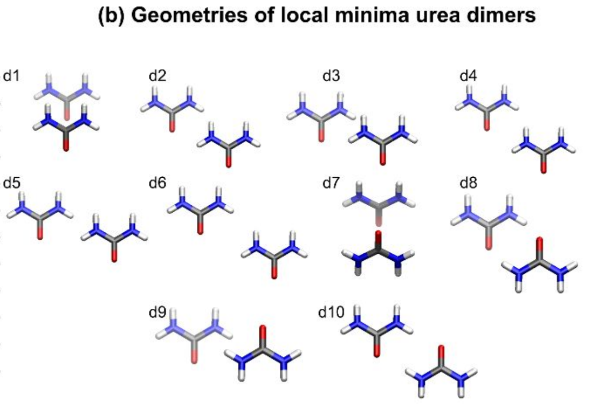

Figure S10. Non-rotated and 180° around y-axis rotated urea local minima dimers, found with REVPBE0/6-311++G/COSMO. Panel (a): Spatial positions of the obtained dimers and their energies. Panel (b): Orthoscopic view on the dimer geometries.

6.2. Benzene dimer search

Benzene dimers are well known in the literature, making them another ideal test case for the current aggregation program. The lattice used to study benzene dimerization took advantage of the rotational symmetry of benzene, which allowed us to restrict our study to rotations around the z-axis between 0° and 60°. Specifically, single point energy calculations were performed at angles from 0 to 90° with steps of 15° for the x and y axes. For the translations along the z-axis, the same values were used as in the coarse grid for porphyrins (from close to twice the Van der Waals radii (3.0 Å) to 5.0 Å with a step of 1.0 Å.), while the translations along the x- and y-axes were determined by the extension of the molecule along its major axes with a step size of 1.0 Å.[6, 9].

Single-point energy calculations for the benzene dimers were conducted using B3LYP/lanl2dz/COSMO. The structure energies are shown in Figure S11. One can identify the sandwich dimer (7), T-shaped dimer (3), parallel-displaced dimer (2)[10], together with other variations of T-shaped dimers (1, 4, 5, 6), also described in the literature[11]. These dimer structures became our starting points for the benzene trimer search.

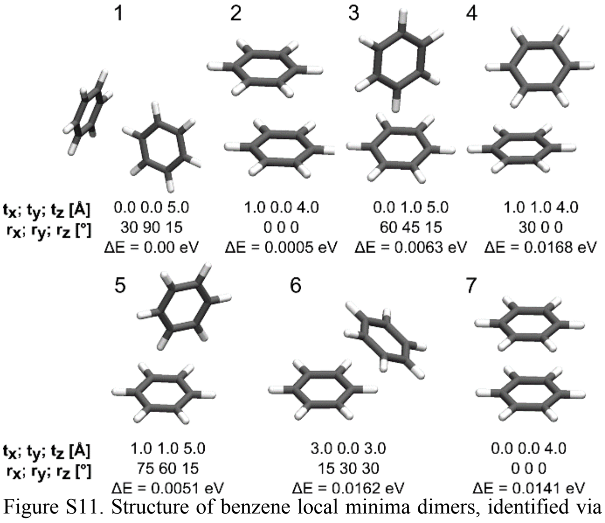

Figure S11. Structure of benzene local minima dimers, identified via B3LYP/lanl2dz/COSMO single point calculations, together with translational vectors and rotations of the second porphyrin, and their energies.

6.3. Benzene trimer search

After determining the dimer structures for benzene according to Figure S11, we used our approach to identify the geometries of benzene trimers, which are also well-known in literature.[6, 9, 12] Therefore, single monomers were scanned with dimer molecules over rotational grid (15° steps from 0 to 90 around x, y and z axes) and coarse translational grid with step 1.0 Å for all axes, displacements over z axes range from 4.0 to 6.0 Å, over x and y axes from 0.0 to 6.0 Å. In Figure S12 the determined local minima structures for different starting dimers (1, 2, 3, 4, 6 as depicted in Figure S11) are presented.

Single-point quantum chemical energy calculations were performed using the B3LYP/lanl2dz/COSMO method, and the local minima search followed the same procedure as used for the dimers. After identifying all local minima structures, they were further optimized using B3LYP/ahlrichs_pvdz/COSMO. After optimization, the trimers were compared in terms of their similarity and energies. The corresponding similarity analysis heatmap, dendrogram and energy ranking are presented in Figure S13, together with the diverse local minima structures found. Among them the most interesting are the global minima trimers 1 and 7, which are both showing the same geometry, known from literature as a cyclic and energetically most favorable structure (labelled "c")[10].

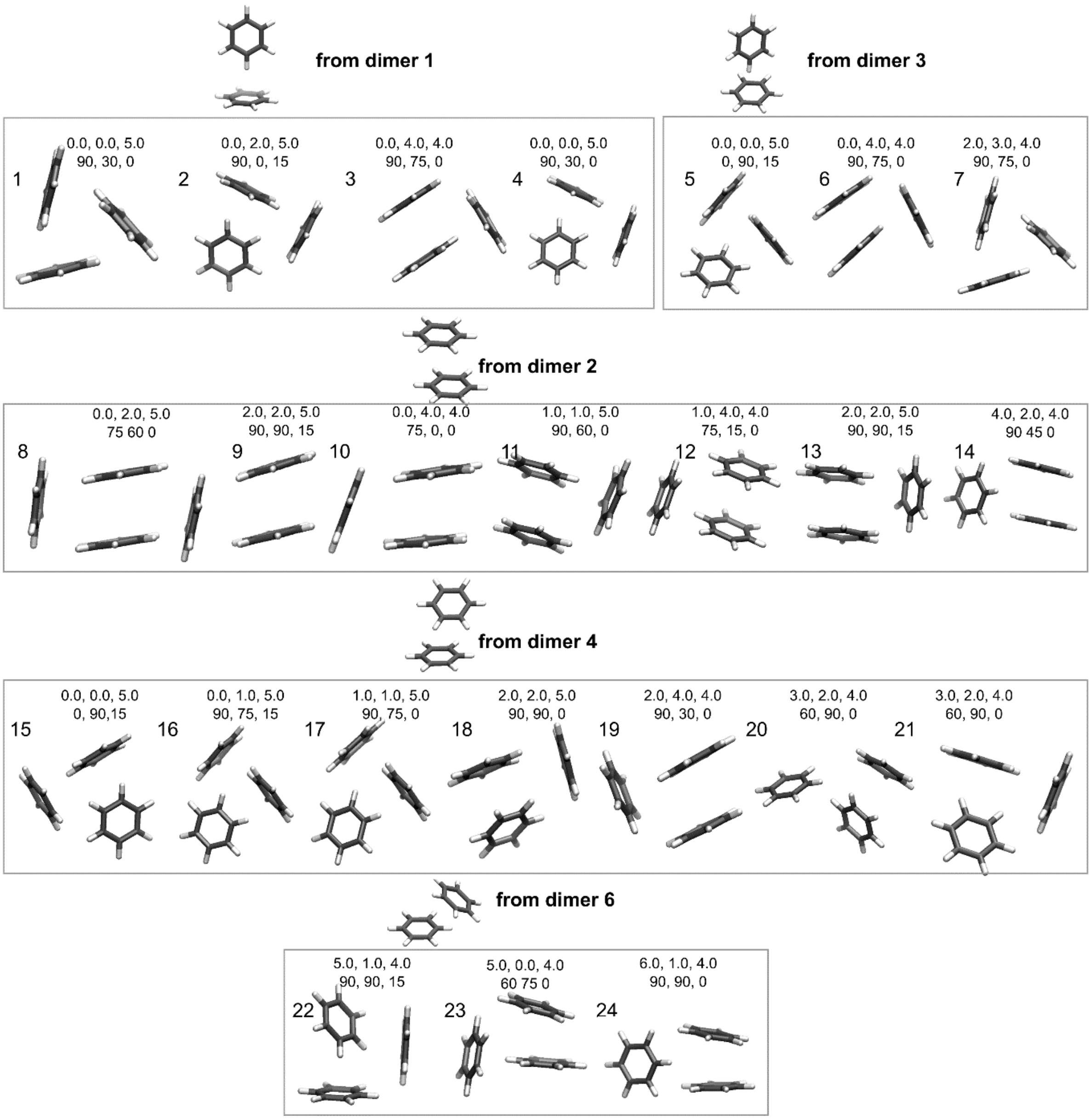

Figure S12. Benzene local minima trimer structures, found with single point DFT calculations using B3LYP/ahlrichs_pvdz/COSMO. The starting dimers are corresponding to Figure S11.

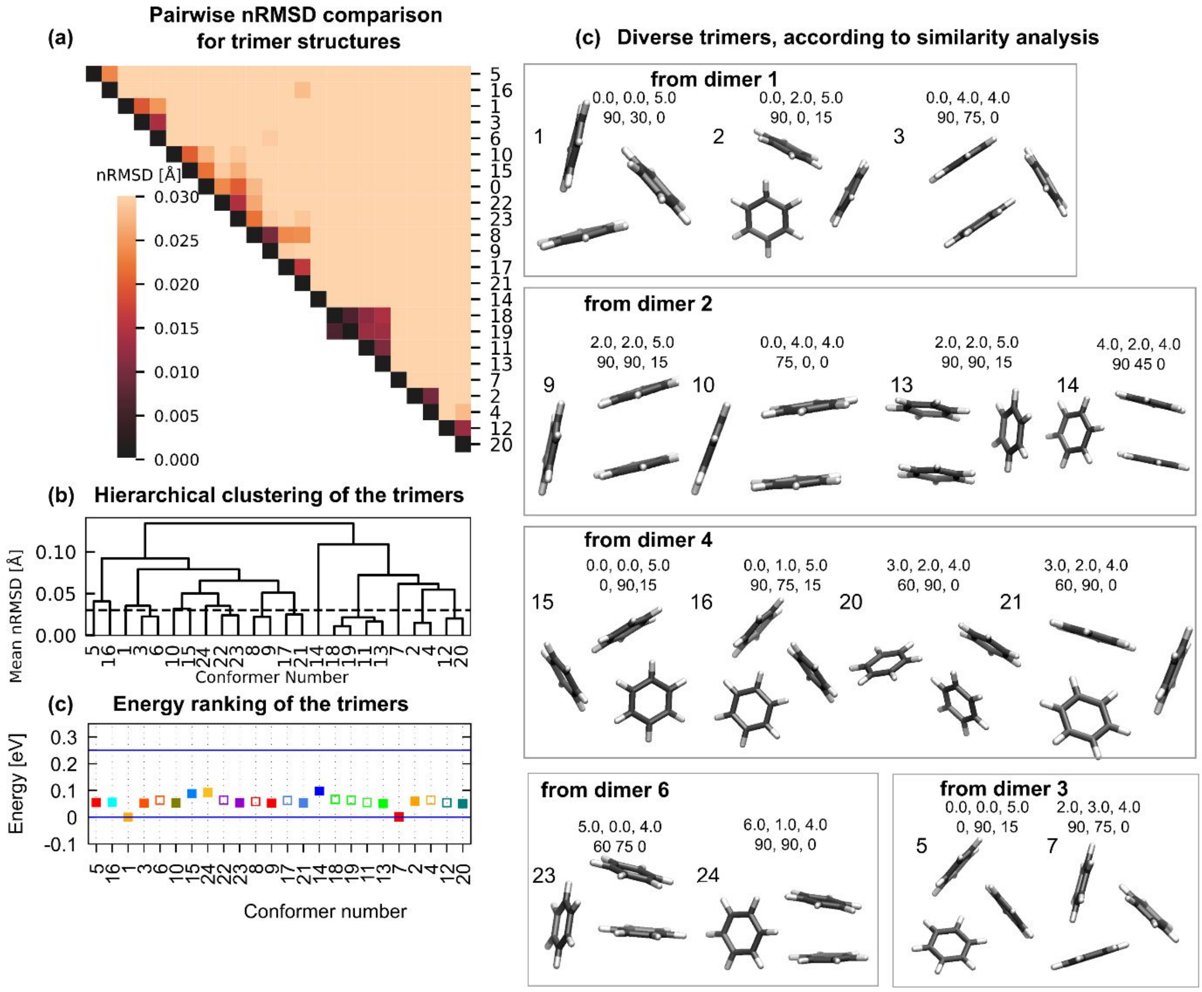

Figure S13. Results of geometrical optimization with B3LYP/ahlrichs_pvdz/COSMO for benzene trimers. Panel (a): Pairwise nRMSD comparison of the trimers. The numbers of the trimers correspond to the numbers in other panels. Panel (b): Hierarchical clustering of the trimers with dashed line showing the clustering threshold of 0.03 Å. Panel (c): Relative energies of the obtained trimers. Filled squares show the energetically favorable members of families from panel (b). Blue lines indicate energy threshold of 250 meV. Panel (c): Diverse structures, obtained after clustering.

However, other benzene trimer prototypes are discussed in literature, although they are less energetically favorable. These are, according to Sinnokrot and Sherrill[10], the linear sandwich trimer (S), linear parallel-displaced trimer (PD) and T-shaped trimer (T), which were not obtained by our initial search due to the short screening distance in z-direction. However, choosing corresponding starting dimers (T-shaped, parallel-displaced and sandwich as presented in Figure S11) and extended grid (15° steps from 0° to 90° around x, y and z axes and coarse translational grid with steps of 1.0 Å for all axes, displacements over z axes from 4.0 to 9.0 Å in case of starting sandwich dimer and from -4.0 to -9.0 Å in case of T and PD-dimers, over x and y axes from 0.0 to 6.0 Å), we were able to find all required trimer structures. Resulting local minima structures, obtained with DFT single point energy calculations using B3LYP/lanl2dz/COSMO are shown in Figure S14. From the figure, it can be seen that sandwich configuration and PD-configuration were among the lowest energy trimers for their groups even without any further optimizations. The T-trimer is also distinguishable without any optimizations, directly from the single point calculations.

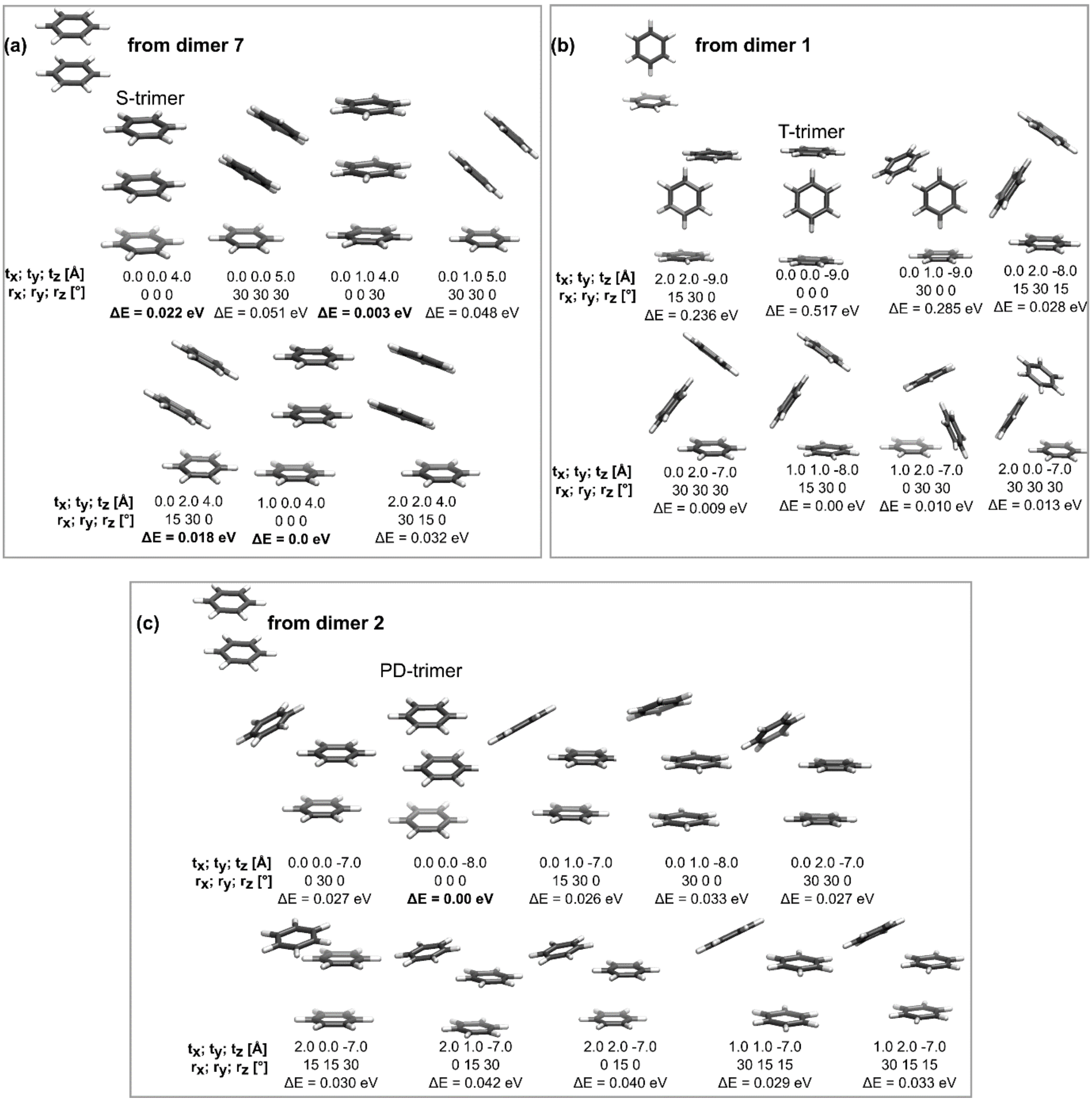

Figure S14. Local minima trimers, obtained with extended over z-axis grids for different starting dimers. Single point energy calculations were obtained with B3LYP/lanl2dz/COSMO. Panel (a): structures, obtained from sandwich dimer, including a sandwich trimer, denoted as S-trimer. Panel (b): Structures, obtained from T-dimer, including the T-trimer, correspondingly denoted. Panel (c): Structures, obtained from parallel-displaced dimer, including the global energetical minimum PD-trimer.

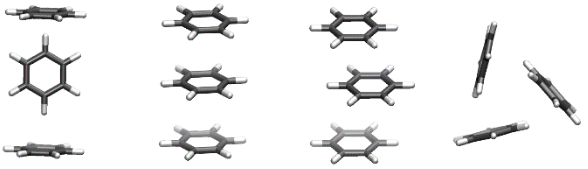

Figure S15. Literature-known benzene trimers, obtained with our algorithm from previously obtained benzene dimers, by scanning a benzene monomer with dimers over rotational and translational grids.

The scalability of the aggregate search procedures employed in this study is promising. The benzene trimers, reported in literature[10] were all successfully found with the employed procedure. By leveraging a systematic approach to local minima identification and structure determination, the method effectively scaled from dimer to trimer configurations, changing from scanning one monomer with another to scanning of a monomer with a chosen dimer structure. The consistency of the results indicates that this approach can be extended to even more complex molecular systems, making it a robust method for exploring larger molecular aggregates. The displacements should be chosen according to the sizes of monomer molecules and their double elongations along different coordinate axes. It is important to notice, that when

scanning a monomer with a dimer, the distances should be calculated not from the monomer, but from the dimer, to avoid molecular clashes.

REFERENCES

(1) Kim, H.; Jang, C.; Yadav, D. K.; Kim, M.-h. The comparison of automated clustering algorithms for resampling representative conformer ensembles with RMSD matrix. *Journal of Cheminformatics* **2017**, *9* (1), 21. DOI: https://doi.org/10.1186/s13321-017-0208-0.

(2) Shao, J.; Tanner, S. W.; Thompson, N.; Cheatham, T. E. Clustering Molecular Dynamics Trajectories: 1. Characterizing the Performance of Different Clustering Algorithms. *Journal of Chemical Theory and Computation* **2007**, *3* (6), 2312-2334. DOI: https://doi.org/10.1021/ct700119m.

(3) Daza, M. C.; Dobado, J. A.; Molina, J. M.; Salvador, P.; Duran, M.; Villaveces, J. L. Basis set superposition error-counterpoise corrected potential energy surfaces. Application to hydrogen peroxide···X (X=F−,Cl−, Br−,Li+,Na+) complexes. *The Journal of Chemical Physics* **1999**, *110* (24), 11806-11813. DOI: https://doi.org/10.1063/1.479166.

(4) Kim, K. H.; Kim, Y. The basis set superposition error in multilevel methods: a test on the H2O and HF dimer. *Theoretical Chemistry Accounts* **2006**, *115* (1), 18-26. DOI: https://doi.org/10.1007/s00214-005-0069-x. Vidal Vidal, Á.; de Vicente Poutás, L. C.; Nieto Faza, O.; Silva López, C. On the Use of Popular Basis Sets: Impact of the Intramolecular Basis Set Superposition Error. *Molecules* **2019**, *24* (20), 3810. DOI: https://doi.org/10.3390/molecules24203810.

(5) Hupfer, M. L.; Kaufmann, M.; May, S.; Preiss, J.; Weiss, D.; Dietzek, B.; Beckert, R.; Presselt, M. Enhancing the supramolecular stability of monolayers by combining dipolar with amphiphilic motifs: a case of amphiphilic push-pull-thiazole. *Physical Chemistry Chemical Physics : PCCP* **2019**, *21* (24), 13241-13247. DOI: https://doi.org/10.1039/c9cp02013f.

(6) Sachse, T.; Martinez, T. J.; Dietzek, B.; Presselt, M. A program for automatically predicting supramolecular aggregates and its application to urea and porphin. *Journal of Computational Chemistry* **2018**, *39* (13), 763-772. DOI: https://doi.org/10.1002/jcc.25151.

(7) Grimme, S. Exploration of Chemical Compound, Conformer, and Reaction Space with Meta-Dynamics Simulations Based on Tight-Binding Quantum Chemical Calculations. *Journal of Chemical Theory and Computation* **2019**, *15* (5), 2847-2862. DOI: https://doi.org/10.1021/acs.jctc.9b00143. Pracht, P.; Grimme, S.; Bannwarth, C.; Bohle, F.; Ehlert, S.; Feldmann, G.; Gorges, J.; Müller, M.; Neudecker, T.; Plett, C.; et al. CREST—A program for the exploration of low-energy molecular chemical space. *The Journal of Chemical Physics* **2024**, *160* (11), 114110. DOI: https://doi.org/10.1063/5.019759. Spicher, S.; Plett, C.; Pracht, P.; Hansen, A.; Grimme, S. Automated Molecular Cluster Growing for Explicit Solvation by Efficient Force Field and Tight Binding Methods. *Journal of Chemical Theory and Computation* **2022**, *18* (5), 3174-3189. DOI: https://doi.org/10.1021/acs.jctc.2c00239.

(8) Stumpe, M. C.; Grubmüller, H. Aqueous Urea Solutions: Structure, Energetics, and Urea Aggregation. *The Journal of Physical Chemistry B* **2007**, *111* (22), 6220-6228. DOI: https://doi.org/10.1021/jp066474n. Masunov, A.; Dannenberg, J. J. Theoretical Study of Urea. I. Monomers and Dimers. *The Journal of Physical Chemistry A* **1999**, *103* (1), 178-184. DOI: https://doi.org/10.1021/jp9835871.

(9) Huber, R. G.; Margreiter, M. A.; Fuchs, J. E.; von Grafenstein, S.; Tautermann, C. S.; Liedl, K. R.; Fox, T. Heteroaromatic π-Stacking Energy Landscapes. *Journal of Chemical Information and Modeling* **2014**, *54* (5), 1371-1379. DOI: https://doi.org/10.1021/ci500183u.

(10) Sinnokrot, M.; Sherrill, C. High-Accuracy Quantum Mechanical Studies of π−π Interactions in Benzene Dimers. *The journal of Physical Chemistry. A* **2006**, *110*, 10656-10668. DOI: https://doi.org/10.1021/jp0610416.

(11) DiStasio, R. A.; von Helden, G.; Steele, R. P.; Head-Gordon, M. On the T-shaped structures of the benzene dimer. *Chem. Phys. Lett.* **2007**, *437* (4), 277-283. DOI: https://doi.org/10.1016/j.cplett.2007.02.034. Cacelli, I.; Cinacchi, G.; Prampolini, G.; Tani, A. Modeling benzene with single-site potentials from ab initio calculations: A step toward hybrid models of complex molecules. *The Journal of Chemical Physics* **2004**, *120*, 3648-3656. DOI: https://doi.org/10.1063/1.1642594. Schnell, M.; Erlekam, U.; Bunker, P. R.; von Helden, G.; Grabow, J.-U.; Meijer, G.; van der Avoird, A. Structure of the Benzene Dimer—Governed by Dynamics. *Angewandte Chemie International Edition* **2013**, *52* (19), 5180-5183. DOI: https://doi.org/10.1002/anie.201300653.

(12) Morimoto, T.; Uno, H.; Furuta, H. Benzene Ring Trimer Interactions Modulate Supramolecular Structures. *Angewandte Chemie International Edition* **2007**, *46* (20), 3672-3675. DOI: https://doi.org/10.1002/anie.200604371. Kennedy, M. R.; McDonald, A. R.; DePrince, A. E., III; Marshall, M. S.; Podeszwa, R.; Sherrill, C. D. Communication: Resolving the three-body contribution to the lattice energy of crystalline benzene: Benchmark results from coupled-cluster theory. *The Journal of Chemical Physics* **2014**, *140* (12), 121104. DOI: https://doi.org/10.1063/1.4869686. Bradeanu, I. L.; Flesch, R.; Kosugi, N.; Pavlychev, A. A.; Rühl, E. C 1s → π* excitation in variable size benzene clusters. *Physical Chemistry Chemical Physics* **2006**, *8* (16), 1906-1913. DOI: https://doi.org/10.1039/B517199G.
